# Major endothelial damage markers identified from hemadsorption filters derived from treated patients with septic shock – endoplasmic reticulum stress and bikunin may play a role

**DOI:** 10.3389/fimmu.2024.1359097

**Published:** 2024-04-18

**Authors:** Robin Kasper, Armando Rodriguez-Alfonso, Ludger Ständker, Sebastian Wiese, E. Marion Schneider

**Affiliations:** ^1^ Clinic of Anesthesiology and Intensive Care Medicine, University Hospital Ulm, Ulm, Germany; ^2^ Core Facility Functional Peptidomics, Ulm University Medical Center, Ulm, Germany; ^3^ Core Unit Mass Spectrometry and Proteomics (CUMP), Ulm University, Ulm, Germany

**Keywords:** septic shock, endothelial damage, hemadsorption, oxidized DNA, bikunin/AMBP, SAA1, CXCL7, HNP-1

## Abstract

**Introduction:**

In septic patients the damage of the endothelial barrier is decisive leading to circulatory septic shock with disseminated vascular coagulation, edema and multiorgan failure. Hemadsorption therapy leads to rapid resolution of clinical symptoms. We propose that the isolation of proteins adsorbed to hemadsorption devices contributes to the identification of mediators responsible for endothelial barrier dysfunction.

**Material and methods:**

Plasma materials enriched to hemadsorption filters (CytoSorb^®^) after therapy of patients in septic shock were fractionated and functionally characterized for their effect on cell integrity, viability, proliferation and ROS formation by human endothelial cells. Fractions were further studied for their contents of oxidized nucleic acids as well as peptides and proteins by mass spectrometry.

**Results:**

Individual fractions exhibited a strong effect on endothelial cell viability, the endothelial layer morphology, and ROS formation. Fractions with high amounts of DNA and oxidized DNA correlated with ROS formation in the target endothelium. In addition, defined proteins such as defensins (HNP-1), SAA1, CXCL7, and the peptide bikunin were linked to the strongest additive effects in endothelial damage.

**Conclusion:**

Our results indicate that hemadsorption is efficient to transiently remove strong endothelial damage mediators from the blood of patients with septic shock, which explains a rapid clinical improvement of inflammation and endothelial function. The current work indicates that a combination of stressors leads to the most detrimental effects. Oxidized ssDNA, likely derived from mitochondria, SAA1, the chemokine CXCL7 and the human neutrophil peptide alpha-defensin 1 (HNP-1) were unique for their significant negative effect on endothelial cell viability. However, the strongest damage effect occurred, when, bikunin – cleaved off from alpha-1-microglobulin was present in high relative amounts (>65%) of protein contents in the most active fraction. Thus, a relevant combination of stressors appears to be removed by hemadsorption therapy which results in fulminant and rapid, though only transient, clinical restitution.

## 1 Introduction

Sepsis is responsible for nearly 20% of all global deaths (1). Thus, it remains one of the most challenging diseases in modern medicine and therefore a major focus of scientific research. The disease is characterized by a complex immune dysfunction, which ultimately leads to endothelial damage accompanied by disseminated intravascular coagulation (DIC), multi organ dysfunction and failure. On the cellular level, the interaction of activated leukocytes and platelets with endothelial cells of the vessel wall is particularly crucial (2–6). During infection, so-called PAMPs (pathogen-associated molecular patterns) such as lipopolysaccharides (LPS), flagellins and others lead to the activation of NF-κB and interferon pathways. Moreover, tissue damage by direct and indirect effects would activate pattern recognition receptors (PRR) with the subsequent release of so-called DAMPs (damage-associated molecular patterns), including non-methylated bacterial and mitochondrial DNA, as well as oxidized lipids, adenosine triphosphate (ATP) and proteins from erythrocyte lysates. Cellular proteins such as high mobility group box 1 (HMBG1), heat shock protein (HSP), ATP, eukaryotic cell-free DNA, apoptotic bodies and metabolites such as uric acid upregulate proinflammatory cytokines. Cytokine release also involves the receptor for advanced glycation end products (RAGE) and toll-like receptor (TLR). As a consequence, more leukocytes are attracted to the inflammatory site of tissue damage resulting in systemic inflammation (4, 7–10). Neutrophils are equipped with various effectors for direct and immediate pathogen defense, such as the release of proteases, particle uptake by phagocytosis and the formation of reactive oxygen species (ROS) such as superoxides and hydrogen peroxides as part of the so-called respiratory burst through NADPH oxidases and superoxide dismutases. Furthermore, the formation of so-called NETs (neutrophil extracellular traps) is a key for pathogen control and inflammation. NETs are conglomerates mostly generated by neutrophils through a process called NETosis. They consist of DNA and antimicrobial proteins such as elastase, myeloperoxidase or cathepsin G (8, 10, 11).

Besides tissue damage by NET formation, major activators of the innate immune system are ROS and reactive nitrogen species (RNS) such as peroxinitrite. Besides NADPH oxidases and superoxide dismutases, as mentioned earlier, ROS are also formed by uncoupling the respiratory chain in mitochondria. They have a procoagulant effect by releasing tissue factor and lead to barrier damage via nitration of cytoskeletal proteins and lipid peroxidation (9).

Many studies have focused on these complicated molecular processes and have shown that hardly a single specific signaling pathway or mediator is responsible for the cellular damage, hyperinflammation and subsequent immune insufficiency and sepsis. Attempts to identify a specific biochemical target and use it for targeted therapy have shown little promise (2, 12). In the last decade, hemadsorption therapy such as CytoSorb^®^ (Cytosorbents, USA) has increasingly come into focus. This procedure aims to remove various cytokines, inflammatory mediators, DAMPs and pathogens from a patient’s blood by binding hydrophobic substances with a molecular weight of up to approximately 60 kDa. Although questioned in a number of recent publications (13, 14), hypercytokinemia appears to respond to hemeadsorption (15). Elimination of the DAMPs intends to help reconstitute the endothelial wall by interfering with signaling pathways that lead to disseminated intravascular coagulation and vessel leakage. The most sensitive components of endothelial cells are the glycocalyx and junctions likely affected by transmigrating leukocytes as well (5).

The aim of the study was to find a common denominator for endothelial stress and endothelial cell viability. Based on the work of Denzinger et al., the focus was set on the main hypothesis that proteins and DNA - are oxidized by ROS during inflammation. These modified proteins may contribute to a scenario of DAMPs leading to cell injury by targeting different pathways of cell death (7).

## 2 Materials and methods

### 2.1 Patient collective

Patients were included in the study if they were admitted to the ICU (G1, Intensive Care Unit) of the University Hospital Ulm within 48 hours due to sepsis and had interleukin-6 (IL-6) levels of more than 500 pg/mL and/or renal failure. Additional inclusion criteria were: Age ≥18 years, need for intensive care, postoperative or posttraumatic condition, SIRS and antibiotic therapy. Patients in neutropenia (<1000/µL), with HIV infection, and pregnancy were excluded. All patients reached septic shock with catecholamine requirement and lactic acidosis (>2 mmol/L serum lactate). Patients were treated following the process guidelines for hemadsorption therapy of the ICU of the University Hospital Ulm. All data collection and specimen collection were performed according to the Declaration of Helsinki and the vote of the Ethics Committee of the University of Ulm (150/16) and with the patient’s informed written consent. According to this study protocol, all hemadsorber devices subjected to biochemical analysis were derived from patients with a positive clinical response.

### 2.2 Isolation of adsorbed proteins

Immediately after the end of hemofiltration treatment, the columns were washed free from blood cells using PBS + 0.005% ethylenediaminetetraacetic acid (ETDA) until the effluent was clear. The contents of 10 hemadsorption devices derived from different septic shock patients were mixed and subjected to detachment of the adsorbed proteins with 50% acetonitrile in water for 1 h. The resin was then centrifuged (4.200 rpm) and the supernatant was filtered (5-13 μm, followed by a 0.45 μm filter). The filtrate was then subjected to ultracentrifugation (1 h and 100,000 xg). The clear supernatant (0.5 L) was diluted 1/10 with water and applied to an ultrafiltration step (cut-off: 30 kDa) to remove albumin. After pooling the samples, the proteins were separated by reversed-phase high performance liquid chromatography (RP-HPLC) at a flow rate of 1.3 mL/min for 40 min. The separated proteins were lyophilized, resuspended in 2 mL of resuspension solution (1% trifluoroacetic acid in water and 5% of buffer containing 80% acetonitrile and 0.1% trifluoroacetic acid in water) and centrifuged at 4,200 rpm for 10 minutes. The supernatant was sterilized by filtration (0.22 µm pore size) and dissolved in cell culture medium. A total of 40 protein fractions was obtained. Protein contents of individual fractions were determined by spectrophotometric methods and adsorption 280 nm; Extinction coefficient of ϵ1% = 6.58 was used (16, 17). Ten percent of the reconstituted filtrate were use in our bioassay. Individual fractions were then characterized on the protein/peptide levels using mass spectroscopy, see chapter 6 below for more details.

### 2.3 Cell culture and endothelial bioassay (confluence, viability and morphology)

Ea.hy926 cells were cultured in Iscove´s modified Dulbecco´s medium (IMDM, Lonza, Switzerland) including 10% fetal calf serum (FCS superior, very low endotoxin, Biochrom, Germany), 3 mM sodium hydrogen carbonate, 25 mM HEPES and 60 µg/mL gentamycin at 37°C and 5% CO_2_. For the bioassay cells were seeded in 96-well microplates at a density of 0.5x10^4^ cells/well. After reaching a confluence of 30%, 10% by volume of the respective protein fractions were added in triplicates along with 2.5 µg/mL propidium iodide (PI, Sigma-Aldrich, USA) to determine cell viability. Long-term live cell imaging (IncuCyte^®^ ZOOM, Essen BioScience, USA) was used to observe the cells for 60 hours and subsequently determine confluence and cell viability. A 20x phase contrast and fluorescence microscope with 565-605 nm excitation and 625-705 nm emission was used for image acquisition. 100 nM staurosporine (STS) was used as a positive control.

Confluence was determined by cell densities and viability was quantified as the red object count per well. To assess morphology, cells were imaged after 50 hours of incubation using the Nikon Eclipse Ts2 phase contrast microscope (Nikon, Japan) using a 40x objective.

### 2.4 ROS formation (MitoSOX ™ Red)

To quantify ROS production in Ea.hy926, cells were seeded at a density of 0.5x10^4^ cells/well in 96-well microplates and cultured until approximately 70% confluence was reached. In triplicates 10% by volume of each protein fraction and 5 µM MitoSOX™ Red reagent (Invitrogen, USA) were added and observed over 6 hours using IncuCyte^®^ ZOOM (20x objective, phase contrast and fluorescence detection with 565-605 nm excitation, 625-705 nm emission). ROS production was quantified by red object count per well. As a positive control, 20 µM vacquinol (Vac) was used (18).

### 2.5 Quantification of oxDNA and DNA

The DNA/RNA Oxidation Damage ELISA kit from Cayman Chemical (USA) was used to quantify oxidized DNA in the collected fractions. This acetylcholinesterase competitive ELISA allows the detection of previously described proteins released by the body through repair processes after oxidation of certain bases (mainly guanine) in the blood and ultimately excreted in the urine, including 8-OHG, 8-OHdG and 8-hydroxyguanine.

Additionally, the DNA content of adsorbed proteins was quantified fluorescently using Quantus™ Fluorometer (Promega, USA). Transparent 500 μL tubes were filled with 20 μL 1x trypsin/EDTA buffer, 80 μL of the respective fraction, and 100 μL of the fluorescent dye diluted 1:200 in trypsin/EDTA buffer. After incubation for 5 min, the samples were measured in the Quantus™ fluorometer.

### 2.6 Peptide/protein analysis

For endogenous peptide analysis, the samples were reduced with 5 mM DTT for 20 min at room temperature and carbamidomethylated with 50 mM iodoacetamide for 20 min at 37°C. The samples were previously run under denaturing conditions on 4% to 20% gradient polyacrylamide gels for protein analysis. A polyacrylamide gel lane section (>10 kDa) was separated, and in-gel digested with Trypsin (ThermoFisher Scientific, USA), at a 1:50 ratio (enzyme:protein) for 16 h at 37°C. The proteolytic fragments were extracted with a mixture of water/acetonitrile (1:1), dried up and then reduced and carbamidomethylated as previously explained.

A 15 µL-aliquot was used for mass spectrometry analysis in an Orbitrap Elite Hybrid mass spectrometry system (Thermo Fisher Scientific, Bremen, Germany) online coupled to an U3000 RSLCnano (Thermo Fisher Scientific, Idstein, Germany) employing an Acclaim PepMap analytical column (75 μm × 500 mm, 2 μm, 100 Å, Thermo Fisher Scientific, Bremen, Germany) at a flow rate of 250 nL/min, as previously described (19). Database searches (PEAKs’ standard workflow: *de novo* + PEAKs DB + PEAKs PTM + Spider) were performed using PEAKs X + studio (20–23). For peptide identification, MS/MS spectra were correlated with the UniProt human reference proteome set (UniProt release 2020_08; 20374 reviewed entries). Parent mass error tolerance and fragment mass error tolerance were at 15 ppm and 0.5 Da, respectively. Maximal number of missed cleavages was set at 3. Carbamidomethylated cysteine was considered as a fixed modification; methionine oxidation, carbamylation and N-terminus acetylation were considered as variable modifications. False discovery rates were set on the peptide level to 1%.

### 2.7 Statistical analysis

SPSS version 26 (IBM, USA) was used for statistical analysis. IncuCyte^®^ ZOOM data were analyzed by one-way analysis of variance (ANOVA) followed by a Bonferroni post-hoc test. The significance level was set at α=0.05.

## 3 Results

### 3.1 Endothelial bioassay – confluence, viability, morphology (IncuCyte^®^ ZOOM)

**Figure 1:**
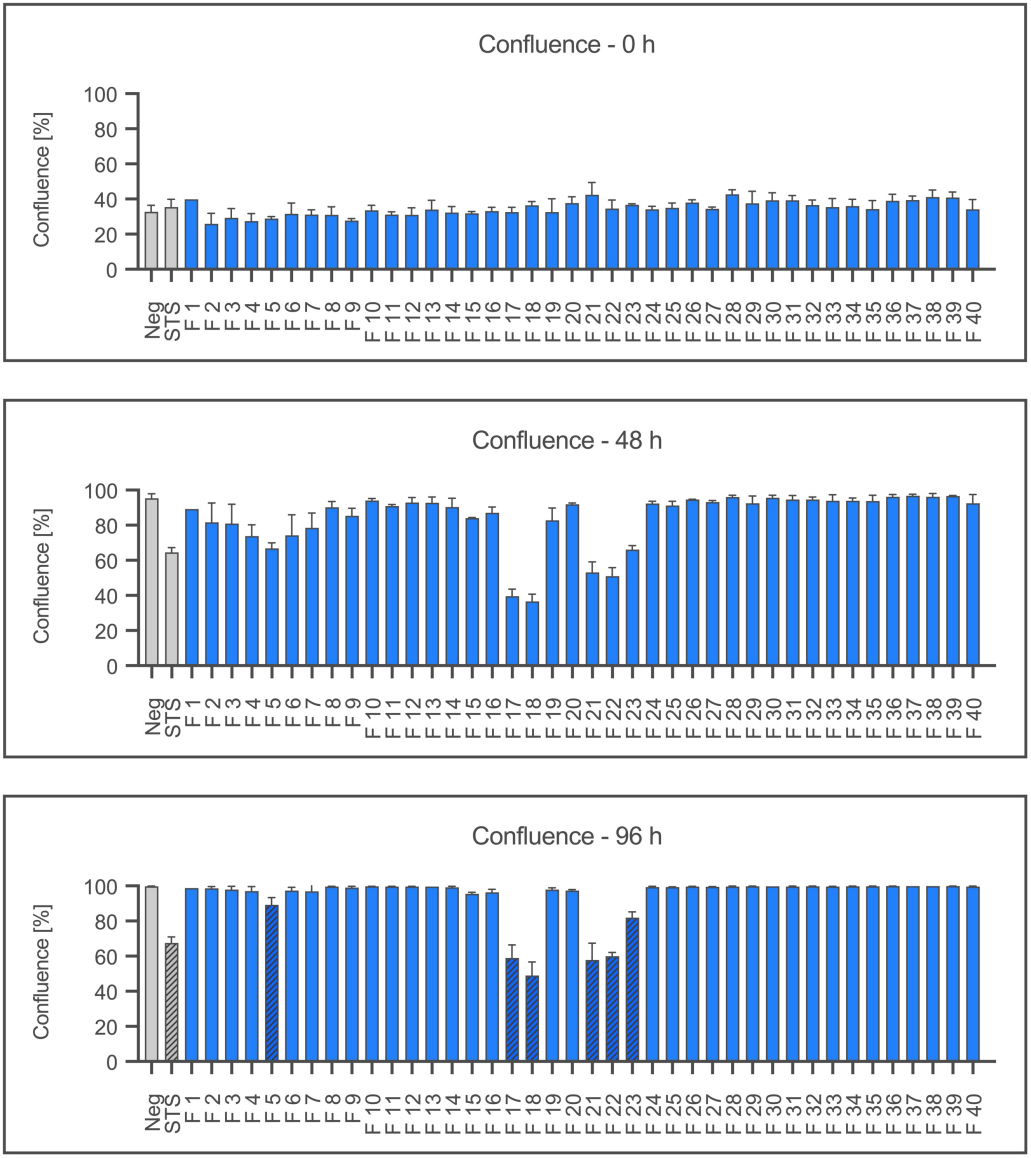
Proliferation of Ea.hy926 cells under the influence of CytoSorb fractions. Shown is the mean confluence and standard deviation of the endothelial cells measured using IncuCyte^®^ ZOOM after 0, 48, and 96 hours in percent. Fractions (F) 1-40, the negative control with IMDM (Negative) and the positive control staurosporine (STS) 100 nM are displayed. Statistical analysis was performed after 96 hours. Significant differences to the negative control are marked as hatched columns.

**Figure 1** shows the mean confluence after 0, 48 and 96 hours (h) of incubation. After 96 h of incubation, fractions 5, 17, 18, 21, 22 and 23 show a significantly reduced confluence, like the positive control STS 100 nM (p ≤ 0.05). Fraction 18 only reached 48.95% of confluence, while the negative control with IMDM attained 100%.

**Figure 2:**
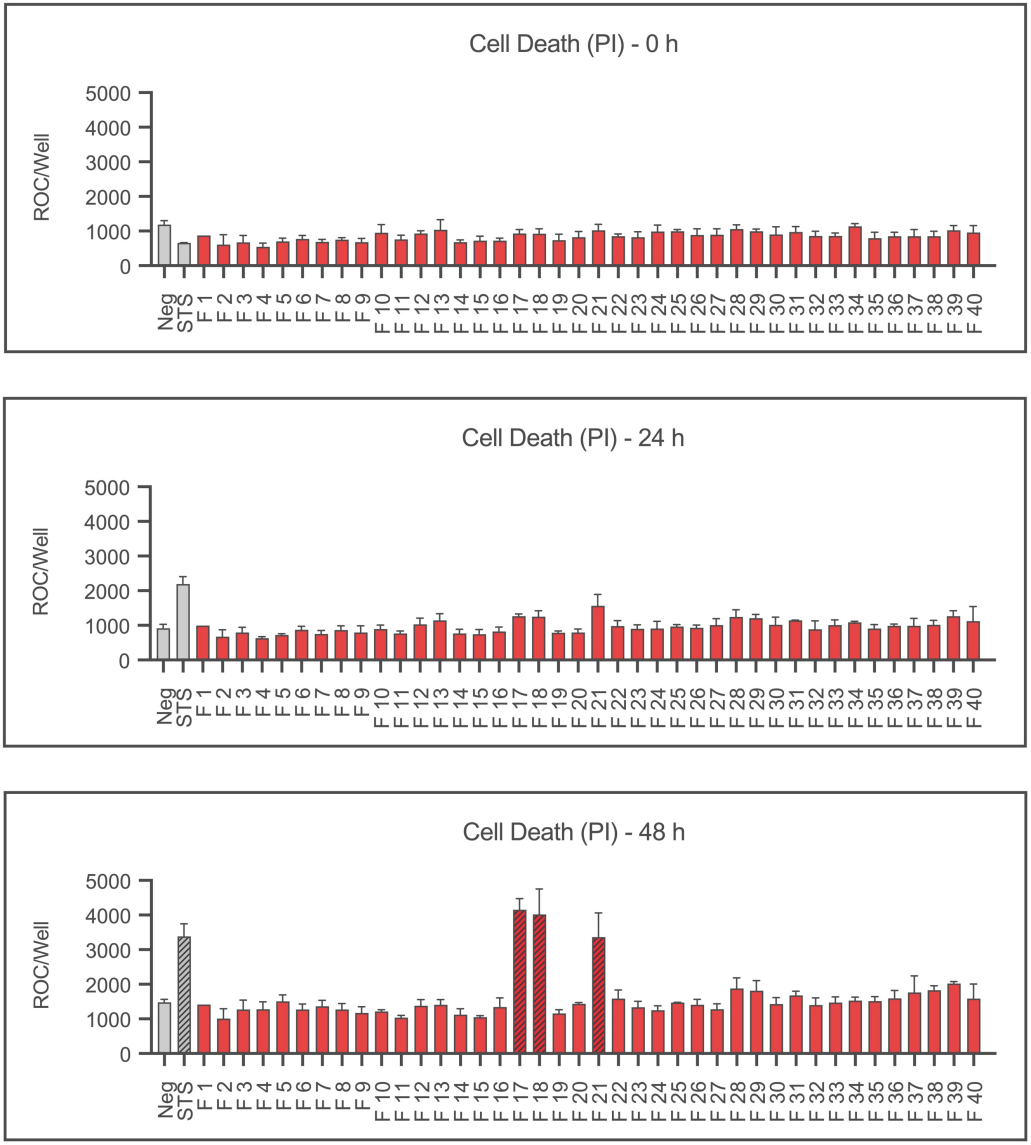
Cell death of Ea.hy926 cells under the influence of CytoSorb fractions. Displayed is the cell death of endothelial cells measured using IncuCyte^®^ ZOOM after 0, 24 and 48 hours as mean red object count per well along with the standard deviation. Fractions (F) 1-40, the negative control with IMDM (Negative) and the positive control staurosporine (STS) 100 nM are shown. Statistical analysis was performed after 48 hours. Significant differences to the negative control are marked as hatched columns.

**Figure 2** shows the cell death as the mean red object count per well after 0, 24 and 48 hours as a result of staining dead cells with propidium iodide. After 48 hours, significantly increased numbers of dead cells are shown by fractions 17, 18, and 21 together with the positive control staurosporine (p≤0.05). Interestingly, fractions 5, 22 and 23 also showed no significant effect on the viability despite severely reducing the confluence (cf. **Figure 1**).

**Figure 3:**
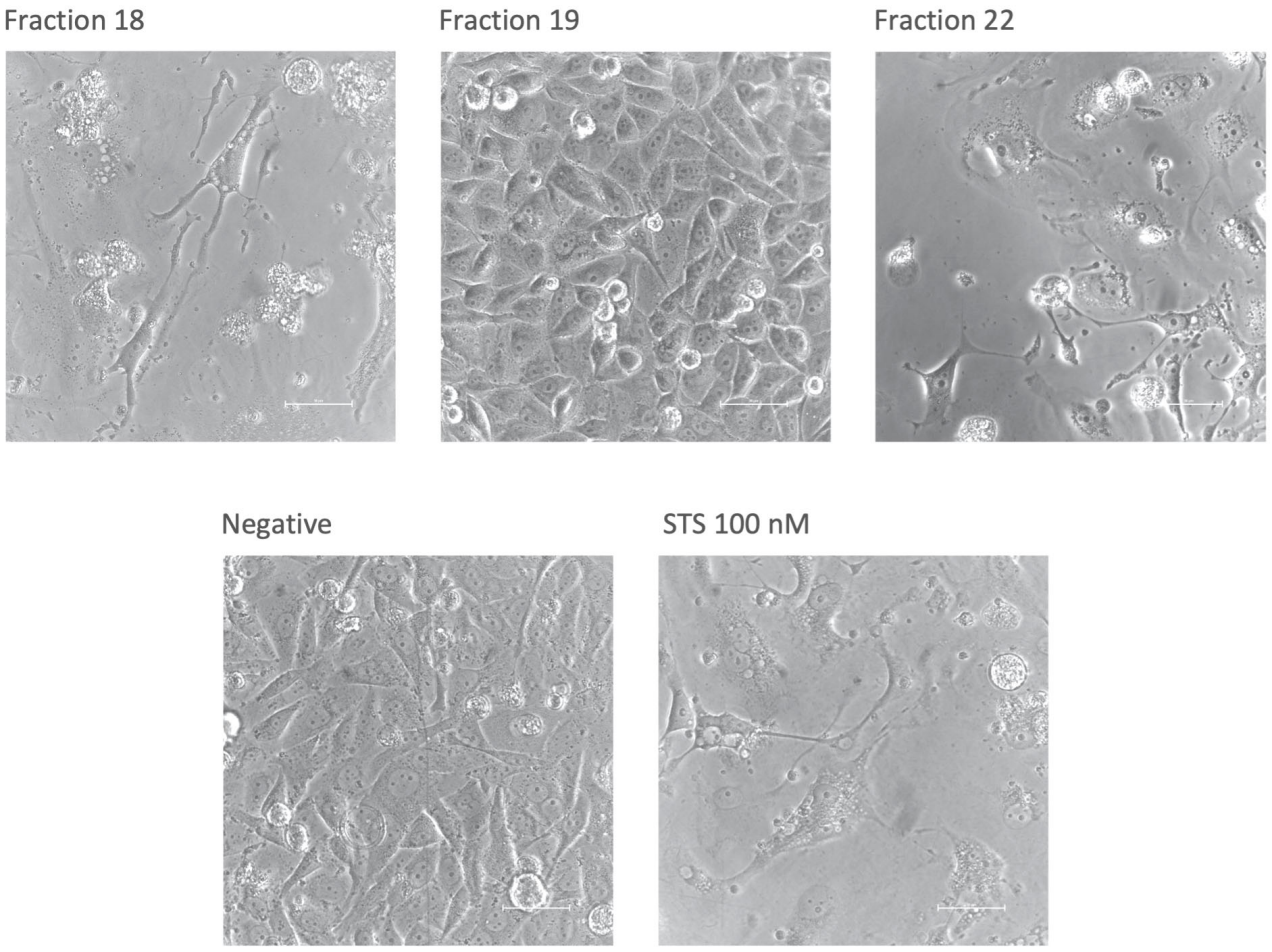
Morphology of Ea.hy926 cells after 50 hours under the influence of CytoSorb fractions. Images were taken with phase contrast microscopy (Nikon Eclipse Ts2, Nikon, Japan) at 40x zoom. Shown are the fractions 18, 19 and 22, the negative control with IMDM (Negative) and the positive control with staurosporine (STS) 100 nM. The scale bar equals 50 µm.

After confluence and viability were assessed, selected fractions were photographed by phase contrast microscopy at 40x magnification to evaluate their morphology. For comparison, fraction 19 is shown here as an example of a regular morphology (**Figure 3**). Fraction 18, which caused a significant decrease in both confluence and viability, appears to induce an apoptotic cell pattern. Notable is the fragmentation of the cells with formation of so-called apoptotic bodies similar to the positive control with staurosporine. Cells under the influence of fraction 22 show a similar appearance, although, despite reduced confluency, viability was not affected.

### 3.2 ROS formation (MitoSOX ™ Red)

**Figure 4:**
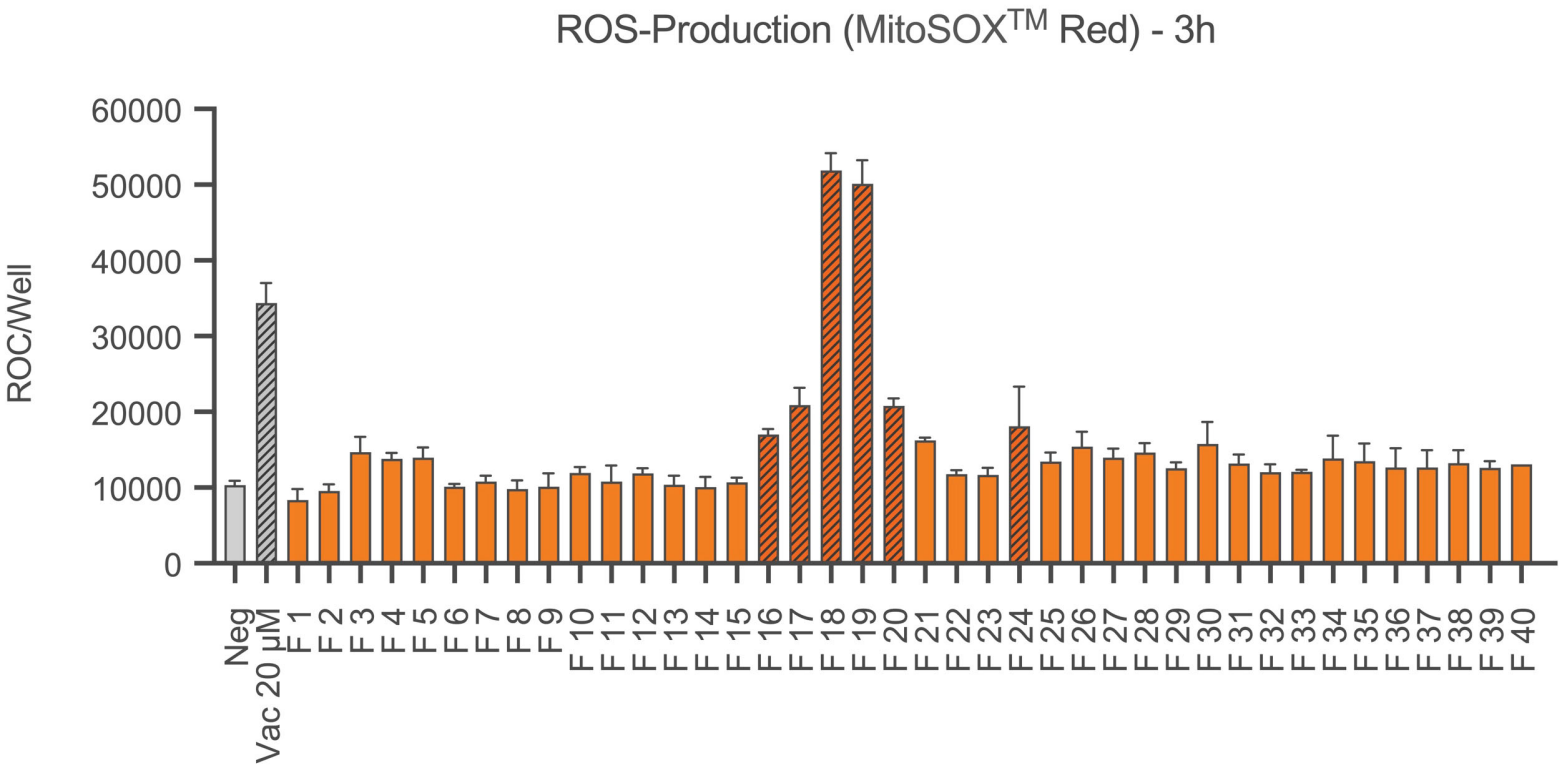
ROS production of Ea.hy926 cells under the influence of CytoSorb fractions. Shown is the production of reactive oxygen species (superoxide anions) as the mean red object count per well along with the standard deviation using MitoSOX™ Red. Quantification was performed with IncuCyte^®^ ZOOM after 3 hours. Results for fractions (F) 1-40, the negative control with IMDM (Negative) and the positive control vacquinol (Vac) 20 µM are displayed. Statistical analysis was performed. Significant differences to the negative control are marked as hatched columns.

For the detection of ROS in the form of superoxide anions in the next step Ea.hy926 cells were stained with MitoSOX™ Red. Results are shown in **Figure 4**. After 3 hours, fractions 16, 17, 18, 19, 20 and 24 showed a significant production of ROS compared to the negative control, with fractions 18 and 19 showing more than twice as much red object counts per well than the other fractions. The positive control Vacquinol 20 µM also caused a significant increase in ROS production.

### 3.3 oxDNA quantification (ELISA)

**Figure 5:**
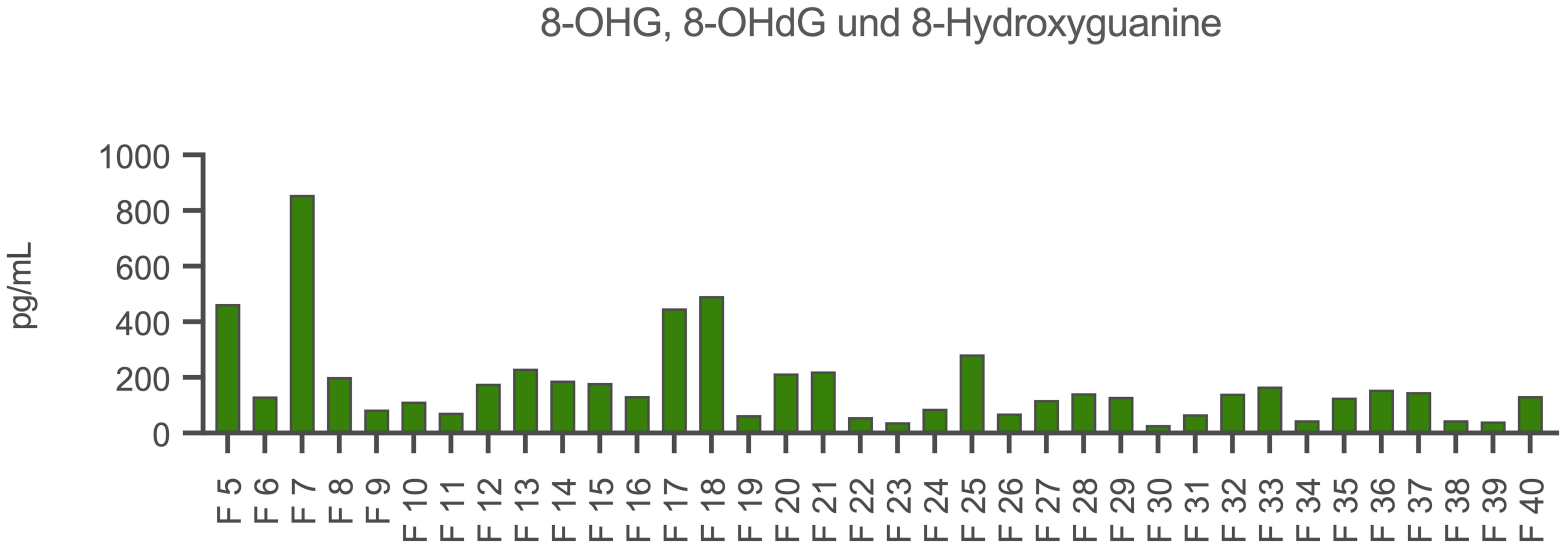
Quantification of oxidized DNA in the CytoSorb fractions. Demonstrated is the content of 8-hydroxyguanosine (8-OHG), 8-hydroxy-2’-deoxyguanosine (8-OHdG) and 8-hydroxyguanine in the fractions (F) 5-40 (x-axis) in pg/mL (y-axis). Measurements were carried out by an acetylcholinesterase competitive ELISA (Cayman Chemical, USA).

The fluorescence-based quantification of dsDNA and ssDNA revealed the highest amounts in fraction 7. Further peaks were found in fraction 18 and for ssDNA also in fraction 24. The results are shown in the **Supplementary Material** (**Supplementary Figure 1**). The amounts of oxidized DNA were then quantified by an acetylcholinesterase competitive ELISA. Accordingly, fractions 5, 7, 17 and 18 contained high amounts of oxidized DNA (**Figure 5**).

Fractions 17, 18, 19, 21 and 22 have shown to be most effective on the endothelial cell layer. To identify protein effectors in the active fractions, we continued with protein and peptide identification experiments.

### 3.4 Peptide analysis of selected fractions 18, 19, 21 and 25

Fractions 18, 19, 21 and 25 were analyzed in greater detail. Using mass spectrometry, the entire peptide and protein profile of these fractions was identified. Fraction 18 was found to be one of the most noticeable fractions across all experiments. As a strong endothelial stressor, it contains high amounts of DNA and oxidized DNA. Fraction 19 also resulted in a very high ROS production but did not show high amounts of DNA nor did it damage endothelial cells compared to fraction 18. Fraction 21 resulted in extensive endothelial damage, although the inducible radical formation was not detected, and its DNA content was low. These observations raised the question of another mediator other than those envisioned from our basic hypothesis. Finally, fraction 25 was selected as a quasi-negative control because it was widely inactive in the bioassay.

**Figure 6:**
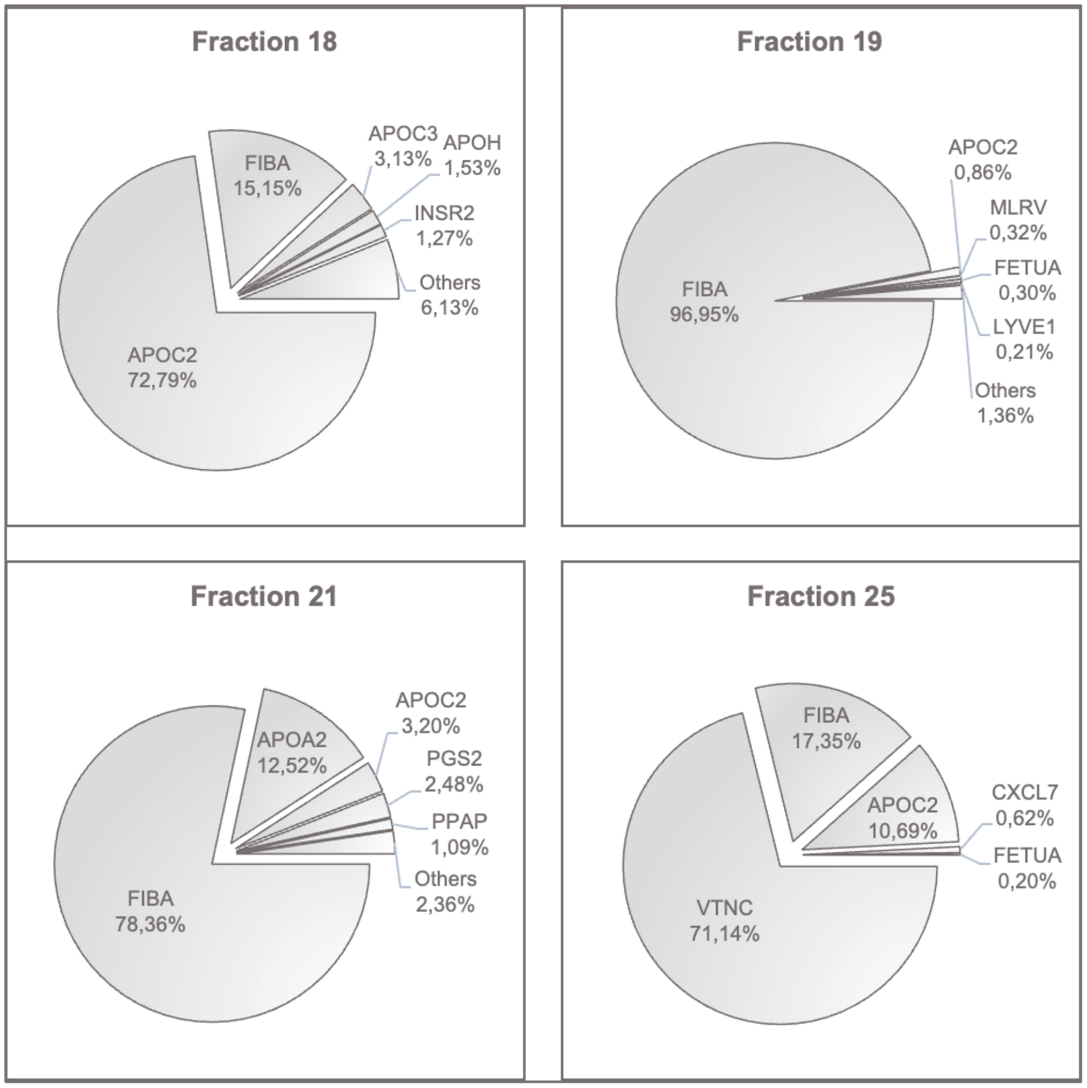
Peptides with the highest contents in fractions 18, 19, 21 and 25. The most prevalent peptides in each fraction are shown in percent of the determined intensities. APOC2, Apolipoprotein C-II; FIBA, Fibrinogen Alpha Chain; APOC3, Apolipoprotein C-III; APOH, Beta-2-Glycoprotein 1; INSR2, Insulin Isoform 2; MLRV, Myosin Regulatory Light Chain 2; FETUA, Fetuin A, Alpha-2-HS-Glycoprotein; LYVE1, Lymphatic Vessel Endothelial Hyaluronic Acid Receptor 1; APOA2, Apolipoprotein A-II; PGS2, Decorin; PPAP, Prostatic Acid Phosphatase; VTNC, Vitronectin; CXCL7, Platelet Basic Protein.

The entire peptide and protein profile of these fractions was identified by mass spectrometry. A rank order comparison of the peptides was made for each fraction. **Figure 6** shows the cake charts of the respective fractions, including the found peptides, identified as their percentage share of the measured intensities. Here, only the most prevalent peptides are shown. The full peptide content of the fractions 18, 19, 21 and 25 as well as post-translationally modified peptides in the form of oxidation are illustrated in the **Supplementary Figures 2**–**5**.

High peptide levels of apolipoproteins and fibrinogen were consistently found in all fractions. The critical fraction 18 additionally contained relevant amounts of hemoglobin alpha, hemopexin, and haptoglobin. This was also found in fractions 19 and 21, respectively. Importantly, fractions 18, 19, and 21 were positive for acute-phase protein SAA1, and in fractions 18, 21 and 25, the platelet activation chemokine CXCL7 was present. Fraction 25 was unique in its vitronectin content.

In summary, this type of analysis detected proteins involved in coagulation, matrix proteins, endothelial receptors, and more constituents of degenerative elements such as Myosin Regulatory Light Chain 2, and proteins bound to chylomicrons and triglyceride-rich low and high-density lipoproteins as well as cholesterol. Fetuin A, detected in fraction 19, indicates the presence of liver-derived factors involved in cardiovascular risk and ectopic calcium homeostasis by its binding capacity of LDL and VLDL receptors. LYVE-1 is a CD44 homologue released from lymphatic vessels after injury. PGS-2, syn. decorin, is another matrix member protein involved in collagen fibril assembly.

### 3.5 Protein analysis of selected fractions 18, 19, 21 and 25

**Figure 7:**
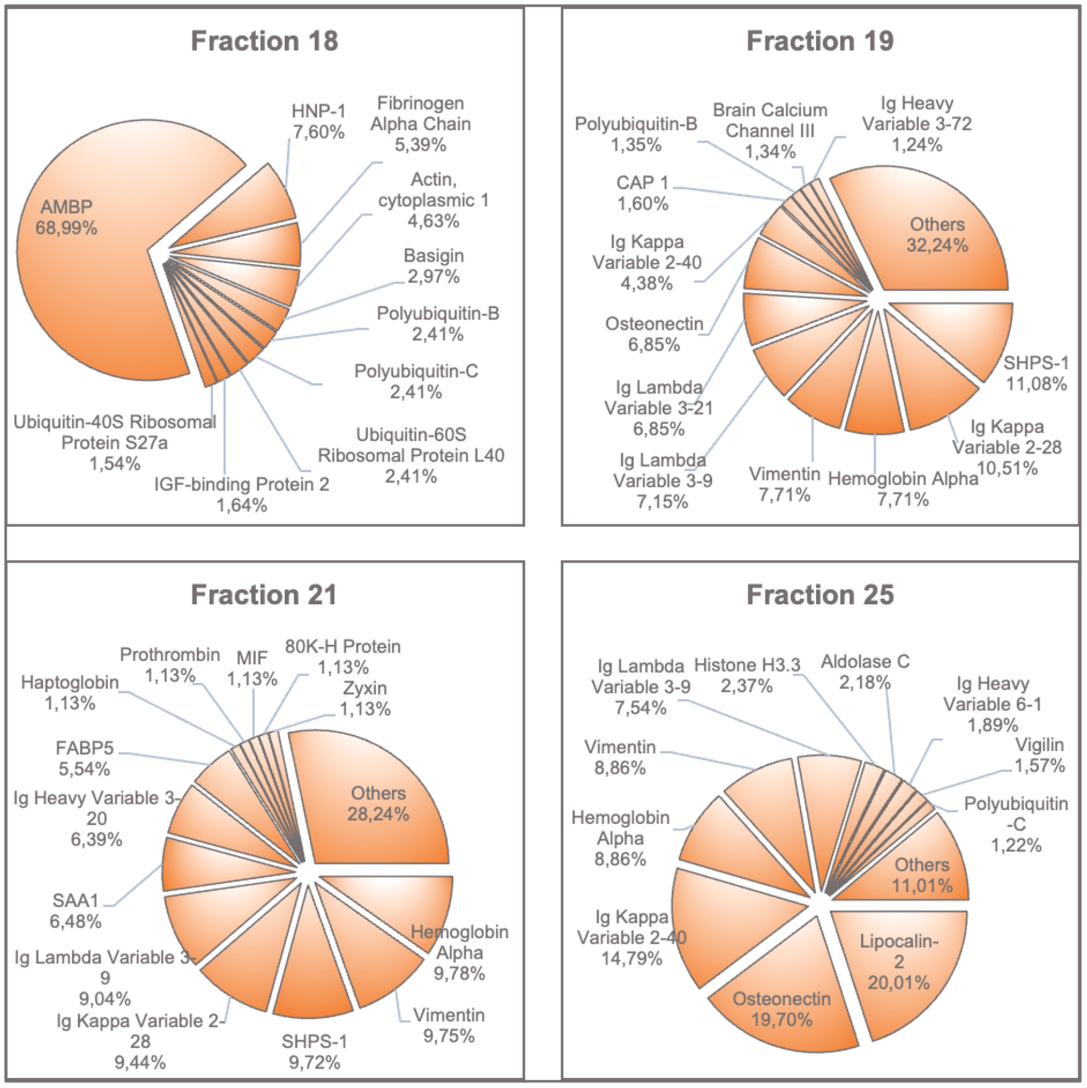
Proteins with the highest amounts found in fractions 18, 19, 21 and 25. The most prevalent proteins in each fraction are shown in percent of intensities. AMBP, Alpha-1-Microglobulin/Bikunin Precursor; HNP-1, Neutrophil Defensin 1; SHPS1, Tyrosine-Protein Phosphatase; CAP1, Adenylyl Cyclase-Associated Protein 1; SAA1, Serum Amyloid A-1 Protein; FABP5, Fatty Acid-Binding Protein 5; MIF, Macrophage Migration Inhibitory Factor; 80K-H Protein, Glucosidase 2 Subunit Beta.

In addition to the peptide profile, the protein profile of fractions 18, 19, 21 and 25 was characterized by mass spectrometry following SDS-PAGE. Due to the large number of proteins found in each fraction, only the most common ones are shown in **Figure 7**. **Supplementary Figures 6**–**9** show the extended results of these protein analyses. The critical fraction 18 predominantly contains the proteins AMBP (alpha-1-microglobulin/bikunin precursor) and HNP-1 (human neutrophil peptide-1, also: alpha-defensin-1). In general, high intensities of vimentin, SHPS-1 (tyrosine-protein phosphatase non-receptor type substrate 1) and the acute-phase protein SAA1 were detected. Unlike the peptide profiles, hemoglobin-alpha accounted for the highest content in fraction 21. A comparable intensity was found in fraction 19, and a higher intensity occurred in fraction 25.

## 4 Discussion

The current report focused on the immediate effects by hemadsorption therapy to relieve septic shock in patients treated by intensive care. An endothelial bioassay and protein chemistry of hemadsorbed materials were applied to identify the most detrimental compounds disrupting endothelial cells. The stepwise approach to first fractionate the hemadsorbed material from a panel of devices from 10 patients, as opposed to a single hemadsorber (7) and test the protein fractions on human endothelial cells rather than rat brain-derived microvascular endothelium as in Denzinger et al. (7), confirmed our previous notion, that a definable composition of molecular entities has the most detrimental effect on endothelial cell layers *in vitro*.

### 4.1 Results from protein analysis of most active fractions 18, 19, 21 and 25

A major component of the most active protein fraction 18 is AMBP (alpha-1-microglobulin/bikunin precursor). Protein analyses identified the AMBP signal as the 16 kDa cleavage product bikunin (also known as inter-alpha-trypsin inhibitor light chain, or ITI-LC for short). The liver is the most important organ to synthesize bikunin from its AMBP precursor protein. AMBP also encodes alpha-1-microglobulin (https://www.ncbi.nlm.nih.gov/gene/259). Maturation of bikunin is associated with proteolytic cleavage of alpha-1-microglobulin and bikunin. The latter is then modified by glycosylation in the Golgi apparatus and further linked to heavy chains through an ester bond with a non-sulfated N-acetylgalactosamine (GalNAc) residue of the chondroitin sulfate (CS) chain. This molecule is released into the plasma (24). A schematic describing the synthesis of AMBP encoded peptides is given in **Supplementary Figure 12**.

Under physiological conditions, the proteoglycan bikunin circulates as an inactive form bound to inter-α-trypsin inhibitor (IαI). IαI family members interact with hyaluronan (HA), inhibit complement, and provide cell regulatory functions (25). IαI molecules from circulation bound to heavy chains (**Supplementary Figure 12**) are transferred to the extracellular matrix HA, thus forming the serum-derived HA-associated protein-HA complex (SHAP-HA), which supports the formation of new ECM and its stabilization (26). In the context of inflammation in sepsis, bikunin may inhibit a number of tissue damaging serine proteases which are released from neutrophils such as elastase or cathepsin G (27). Moreover, bikunin may inhibit plasmin as well as hyaluronidase and may thus attenuate neutrophil activation (25). Generally, the effects by bikunin are difficult to separate from the alpha-1-microglobulin and the IαI-bound complex, unless an alpha-1-microglobulin knock-out model is applied. This has been carried out by Bergwik and colleagues (28). Here a profound pro-inflammatory effect with endothelial damage has been described as well as fatty acid induced liver damage likely mediated by ER-stress (28). These effects are aggravated by the fact that alpha-1-microglobulin has an antioxidant effect on the organism due to its reductase activity (29). The protective effect by enhanced expression of alpha-1-microglobulin during oxidative stress further leads to better cell uptake and binding to complex I of the respiratory chain to maintain ATP production and prevent cell swelling (30, 31).

Chondoitinase-treated bikunin results in a reduced molecular weight compound with around 25-26 kDa when compared to chrondroitin-bound bikunin with 38 kDa (32). The authors further argued that „Possibly, free bikunin has an inflammation-related function and its normal plasma concentration should therefore be low, as reflected by its rapid turnover.” (32). The anti-oxidant and tissue protective functions are clearly linked to the inter-alpha-inhibitor protein (IαIP) not bikunin as shown by Htwe and colleagues (33) (cf. **Figure 4** as an example). The authors here determined neutrophils shape changes and summarized: “IAIP, but not bikunin, maintains spherical shape, small size, and smooth surface of human neutrophils and supports capillary passage; IAIP reduced ROS production from neutrophils in a concentration dependent manner, probably through p47phox phosphorylation” (33). According to Kaczmarczyk et al., bikunin is quickly removed from circulation (approximately 7 minutes) not to interfere with tissue reconstitution, which is why excretion via urine as urinary trypsine inhibitor (UTI) or ulinastatin is highly relevant and plasma concentrations do not exceed 5 µg/mL (22). Inflammatory conditions described significant modifications of the bikunin side chains regarding both sulfation and chain length composition of the CS moiety (34). Accordingly, free bikunin has been termed a novel acute phase protein (24). The inhibitory action of bikunin on endothelial cell layers is likely due to the interference with the calcium-dependent transforming growth factor-beta 1 signaling cascade (35). Acquired as well as inherited proteoglycan biosynthesis defects may account for loss of tissue protective effects by bikunin, as described in various forms of the Ehlers Danlos Syndrome linked to deficient synthesis of glycosaminoglycan (36). Thus bikunin identified by altered chondroitin side chains has been identified as a biomarker and acute phase protein (37), see schematic in **Supplementary Figure 12**.

To test our hypothesis of cytotoxic effects by free bikunin, we performed another series of bioassays with Ea.hy926 endothelium. For this purpose, the cells were incubated for a total of 72 hours with bikunin isolated from human urine at concentrations of 7 and 70 µg/mL. Dead cells were detected by propidium iodide staining, all nuclei were stained with Hoechst33342 and the Live Cell Imaging’ system JuLI™ Stage (NanoEnTek.com) was applied for monitoring. Despite the lack of significance, a dose-dependent cytotoxic effect of bikunin on endothelial cells was observed (**Supplementary Figures 10**, **11**).

Another protein of interest in fraction 18 is HNP-1 (Human Neutrophil Peptide-1, syn. Neutrophil defensin 1) which was high in fraction 18 and only found in small amounts in fraction 25. HNP-1, along with HNP-2, HNP-3 and HNP-4, is a member of the α-defensin group which is mainly expressed by neutrophil granulocytes and stored in intracellular vesicles. HNP-1, HNP-2 and HNP-3 have a strong antimicrobial effect by binding as cationic molecules to the enhanced negatively charged phospholipid membranes of bacteria after fusion with the phagosome, causing pore formation (38–42). In sepsis, increased gene copy numbers for HNP1-3 were associated with higher susceptibility to a severe outcome (43). Further studies demonstrated that this increased gene copy number in the mouse model relates to endothelial barrier damage with subsequent organ dysfunction. Chen and colleagues demonstrated the induction of pyroptosis by HNP-1 in murine lung microvascular endothelial cells. Binding of HNP-1 to the so-called P2X7 receptor (purinergic receptor P2X ligand-gated ion channel 7), a PRR, activates the canonical caspase-1 via the NLRP3 inflammasome (44). Caspase-1 further cleaves gasdermin D, giving it the ability to destroy the cell by pore formation and thus plunging it into pyroptosis (45).

The overall protein concentration as well as the abundance of defined proteins in Cytosorb fractions 17, 18, 19, 21 and 25 are shown in the **Supplementary Tables 1**–**6**. Based on the spectrophotometric measurements at 280nm, the protein concentrations of Cytosorb fractions were calculated (**Supplementary Figure 13**).

Fraction 17 was included in these analyses because its cytotoxicity against endothelial cells was similar to fraction 18. The protein profile of fraction 17 revealed relevant amounts of bikunin confirming our assumption of a direct or significantly cooperative cytotoxic effect (**Supplementary Figure 13**).

### 4.2 The role of ROS and DNA

As discussed further, the endothelial damage effect by components of fraction 18 are related to the action of bikunin and HNP-1 but also oxidative stressors play an important role by DAMP-PRR-interactions. In the context of septic shock, most important stressors arise from oxidized DNA, related to NETosis and ROS generated in neutrophils. However, the present investigation shows that i) ROS formation alone does not cause damage to the cells. This notion is based on results obtained from fraction 19 with the highest mitochondrial ROS formation but no significant effects on the endothelial cell layer; ii) ssDNA, likely arising from mitochondria, and dsDNA do not constitute major damage effectors due to negative effects by fractions 6 and 25.

However, the combined action of mitochondrial ROS formation in addition to high DNA contents appears to manifest a significant endothelial damage effect, as exemplified by fractions 18 and 19. When incubating endothelial cells with fractions 18 and 19, ROS formation is strong after only 3 h of co-incubation. Fraction 18 is unique by performing consecutive cell damage with final endothelial cell death from 24 h co-incubation onwards. In addition, fraction 18 contains almost twice as much dsDNA and ssDNA when compared with fraction 19. In summary, endothelial damage is likely to occur by oxDNA however only in combination with other mediators. Levels of oxidized DNA are extremely high in fraction #7 but this fraction lacks a significant damage response. However, our previous analysis also stressed the role of oxDNA as a relevant DAMP (7). The likely oxidation of mitochondrial DNA enriched in the ssDNA fraction occurs by superoxide formation within the mitochondria itself (9). In case of high relevance of ROS and DNA as endothelial stressors, anti-oxidant interventions as well as blockade of signaling by oxDNA should have a significant effect on outcome, lactate and stay on ICU in patients with septic shock (46, 47).

Importantly, hemadsorption was able to remove the oxidative stressor. Superoxides, such as O_2_^-^, are formed by an uncoupling of the respiratory chain in the mitochondria. Physiologically, superoxides are neutralized by antioxidant enzymes requiring NADPH (48). However, NADPH is depleted in the septic organism due to the increased formation of superoxide radicals, the so-called “respiratory burst”, caused by upregulation of membrane-bound NADPH oxidases (9). Thus, in addition to inhibiting radical detoxification, these events would result in an escalation of radical formation and consequently increase oxidation of cell-free DNA. Increased concentrations of 8-OhdG, which have been previously described in septic patients, affect endothelial cell viability (7, 49). Due to its naked nature, mitochondrial DNA is much more likely to be oxidized as compared to eukaryotic DNA and is highly enriched in 8-OhdG, which then activates STING (stimulator of interferon genes) by acting as a DAMP for cGAS (cyclic GMP-AMP synthase) (50). Activation of the cGAS pathway increases interferons as well as proinflammatory chemokines and cytokines as members of the innate immune defense (50). In addition, inflammasome activation by binding of oxidized DNA to RAGE (receptor of advanced glycation end products) must be considered (51, 52). This context has been discussed previously due to a significant correlation between amounts of oxDNA and RAGE from fractions isolated from a Cytosorb hemofiltrate (7). Results for the central role of oxDNA are supported by the observation that oxDNA may lead to an upregulation of NADPH oxidase 4 with increased ROS-production in HUVECs (53). Another pathway induced by mtDNA and its oxidized metabolites may lead to activation of the toll-like receptor 9 (TLR9) with subsequent release of inflammatory mediators. This receptor of the innate immune system recognizes pathogens by binding unmethylated CpG dinucleotides present in the DNA of the pathogen (54–57). According to the endosymbiotic theory of mitochondrial origin, this mechanism would also explain mtDNA effects, especially since TLR9 cooperates with the pathogen receptor TLR4 upon internalization (58). In critically ill patients, levels of mtDNA correlate significantly with mortality in patients having high expression of TLR9 (59, 60). Elevated blood mtDNA levels in patients with sepsis or septic shock have been proposed to serve as a biomarker for sepsis (61–63).

### 4.3 Results from peptide analysis of fractions 18, 19, 21 and 25

In general, the highest signal intensities were generated from fragments of APOC2 (apolipoprotein C-II) and FIBA (fibrinogen alpha chain). During sepsis, lipid metabolism undergoes significant changes. However, APOC2 usually is diminished in this condition (64). Therefore, its involvement as a direct mediator of endothelial cell damage seems unlikely. The reason for the high levels measured could be due to parenteral high caloric nutrition of the patients. FIBA is one of three components of fibrinogen. Besides forming the nd of the physiological coagulation cascade by cross-linking aggregated platelets to form a white thrombus and contributing to the development of a disseminated intravascular coagulation (DIC) in the context of the sepsis, high fibrinogen levels are also considered to be a risk factor for cardiovascular diseases and a biomarker for inflammation (65–67). Tyagi and colleagues demonstrated that fibrinogen in pathologically elevated amounts leads to a disruption of an endothelial cell monolayer with an increase in permeability via ERK (extracellular signal regulated kinase) and F-actin formation (68). Yu and colleagues, on the other hand, demonstrated that fibrinogen, in the form of Fresh Frozen Plasma, has an anti-apoptotic effect on endothelial cells *in vitro* (69). Ultimately, the effect appears to be highly dependent on the clinical condition of an individual patient and to proceed via several potential mechanisms and targets, as summarized by Luyendyk et al. in their review (70).

Fraction 18 was the only one to contain free Hb or its alpha subunit respectively. Furthermore, relevant amounts of Hpx (also present in fraction 19) and Hpt (also present in fraction 21) were found. This observation supports our previous hypothesis, that free Hb is involved in the demise of endothelial cells by acting as another DAMP (7). In contrast, fraction 21, which was also damaging, did not contain Hb, supporting the suggestion that a combination of DAMPs affects vascular integrity. Free heme induces the expression of adhesion molecules via TLR4 in endothelial cells (71, 72). Further, it has been shown that heme, but also Hb, leads to dissolution of the endothelial monolayer and thus contributes to cell extravasation (73–77). Involved in this process are the p38/hsp27 pathway and TLR4-dependent ROS production involving the p38 pathway (74, 76). Heme also led to an activation of the inflammasome via NLRP3 with subsequent IL-1β production in endothelial cells similar to macrophages (78, 79). It was shown that elevated Hb levels were associated with increased mortality as well as increased risk of organ dysfunction (80–85). Red blood cell lysis is triggered by a wide variety of pathways: Disseminated intravascular coagulation, reduced capillary flow, restriction of glucose transport into erythrocytes, alterations of cell membrane properties, hemolytic pathogens, and red blood cell apoptosis (eryptosis) (86). In conclusion, increased release of Hb may contribute to endothelial cell damage and the pathogenesis of sepsis.

Similar to our previous investigation (7), we here confirm the detection of the acute phase protein SAA1 in fractions 18, 19, and 21. This protein is released during inflammatory processes by activation of the NF-kB pathway via TLR4 and RAGE, resulting in the release of the proinflammatory alarmin HMGB1 (87, 88). SAA1 also drives ROS production in endothelial cells and leads to endothelial dysfunction via inhibition of eNOS (89).

Another interesting peptide is CXCL7 found in fractions 18, 21, and 25. This chemokine, which is also known as PBP (Platelet Basic Protein), NAP-2 (Neutrophil Activating Peptide 2) or Beta-TG (Beta-Thromboglobulin), is released in large amounts following platelet activation (90, 91). A trigger for this may be, for example, damage to the vascular endothelium. Via its receptor CXCR2 (CXC chemokine receptor 2), CXCL7 leads to neutrophil recruitment to the site of injury, which in turn promotes the inflammatory reaction (92). An important question in the context of our study is whether this platelet activation was brought about by the sepsis associated pathology, or whether platelet activation occurred from collateral damage by CytoSorb treatment. However, in another study, reduced amounts of CXCL7 (PBP) have been detected after Cytosorb treatment in a lung transplantation model, supporting effective clearance of CXCL7 by this hemadsorption procedure, which favors the sepsis-associated pathology by CXCL7 (93).

Most importantly, some fractions identified here mediated endothelial damage independently from an effect on mitochondrial ROS and with negligible amounts of DNA or oxDNA. These fractions 21 and 22, show a strong influence on the endothelium. Both fractions affect endothelial confluence, but only fraction 21 mediates endothelial cell death. This finding further strengthens the assumption, already explained at the beginning, that possibly not one but an ensemble of multiple mediators ultimately causes cell damage with subsequent organ failure.

In conclusion, the endothelial damage effect by components of fraction 18 are related to the action of bikunin, HNP-1 and oxidative stressors (oxidized and mitochondrial DNA) through DAMP-PRR-interactions. This investigation identified hemadsorbed DNA and protein effector molecules inducing endothelial stress in patients with septic shock. A major limitation of this study is related to the lack of signaling analysis by individual compounds as well as their combinations in the endothelial bioassay. Such studies would provide a more solid knowledge as to whether hemadsorption needs to be either intensified or attenuated. In the case of CXCL7, a higher adsorption capacity might be recommended due to the observations in an ex-vivo lung transplantation model (93). In the case of the current study, blood samples taken from individual patients before and after hemadsorption should be subjected to a similar mass spectrometric analysis. For future experiments, the hemadsorbed protein fraction could be tested in a mouse model or in organoids, which would be superior to unravel organ specific endothelial damage. Before approaching this experimental opportunity, artificial intelligence approaches including cluster analysis of the peptide contents of each fraction isolated, will be realistic to preselect compositions of proteins with and w/o oxDNA for a major effect on endothelial function.

### 4.4 Limitations of the current study

A limitation to the presented approach are possible matrix effects in samples with a huge disparity in protein abundances, such as plasma. The presence of high amounts of Fibrinogen may limit the ability of the mass spectrometer to effectively sample low abundant peptides. In addition, the type of approach undertaken in the present manuscript is further complicated, as highly abundant peptides with a medium activity might actually hide much stronger molar activities of less abundant peptides. Nevertheless, this study succeeded to identify protein entities adsorbed by hemadsorption from patients in septic shock which correlate with a detrimental effect in our endothelial bioassay. However, so far, only protein fractions with different constituents mediated a defined effect on endothelial bilayer’s disruption and cell death. In addition, the proteins non-specifically eliminated by hemadsorption have not been studied. Therefore, future work needs to focus on the mass spectroscopically based analysis of plasma proteins before and after hemadsorption. Preliminary analysis indicates that this approach is relevant to distinguish patients with vs. without clinical improvement after hemadsorption (data not shown). Since recently, delineation of the intracellular pathways regulating endothelial permeability emphasized the unfolded protein response pathways with various protein mediators involved, showed that a positive outcome of ER activation involved global ATF6-mediated protection against disease (94). The morphological observations in our endothelial bioassay showed that ER-stress, vacuolization, and cell blebbing is a likely pathway active in endothelial dysfunction. This ER stress can be targeted and counter regulated by plasma proteins, which might lead the way to substantial post hemadsorption treatment protocols.

## 5 Conclusion

Defined proteins extracted from CytoSorb hemadsorption devices used in septic patients were tested for their effects on endothelial cell death, the formation of ROS, as well as proliferation and morphology of the endothelial cell layer. The most dramatic effect on endothelial cell layer disruption was mediated by a unique protein fraction containing high amounts of bikunin. The mechanism of action needs further investigation to decide whether sepsis modifies bikunin’s sulfation and chain length composition of the CS moiety and whether loss of hyaluronic acid mediated glycocalyx stabilization interferes with calcium signaling and TGF-β signaling. Other fractions with endothelial damage activity emphasize oxidative stress from oxidized nucleic acids, including histones, likely functioning as major DAMPs for the endothelial bilayer. In addition, identification of the protein and peptide profiles of the critical endothelial damage fractions were: Pore forming peptides such as HNP-1, acute phase proteins, such as SAA1, the chemokine CXCL7, and free hemoglobin derivatives.

Importantly, the results confirm the complexity of effector peptides for the pathogenesis of endothelial cell damage in septic shock. The here described endothelial bioassay proved to be sufficient to generate reliable data sets to quantify cell damage in a defined time window and is further suitable to test therapeutically valuable approaches aiming at the reconstitution of the endothelial glycocalyx and microvascular integrity.

## Data availability statement

The original contributions presented in the study are included in the article/**Supplementary Materials**, further inquiries can be directed to the corresponding author/s.

## Ethics statement

The studies involving humans were approved by Ethics committee Ulm University. The studies were conducted in accordance with the local legislation and institutional requirements. The participants provided their written informed consent to participate in this study.

## Author contributions

RK: Investigation, Writing – original draft, Data curation. AR-A: Data curation, Formal analysis, Investigation, Methodology, Software, Writing – review & editing. LS: Investigation, Methodology, Resources, Writing – review & editing. SW: Data curation, Formal analysis, Methodology, Software, Writing – review & editing. ES: Conceptualization, Investigation, Methodology, Project administration, Resources, Supervision, Writing – original draft.
