# Supplementary material for: Major endothelial damage markers identified from hemadsorption filters derived from treated patients with septic shock – endoplasmic reticulum stress and bikunin may play a role
Source: Front Immunol. 2024 Apr 18;15:1359097. doi: 10.3389/fimmu.2024.1359097 (PMC11063272; doi:10.3389/fimmu.2024.1359097)
Supplement: Supplementary file 1 [file DataSheet_1.pdf]

Supplementary material

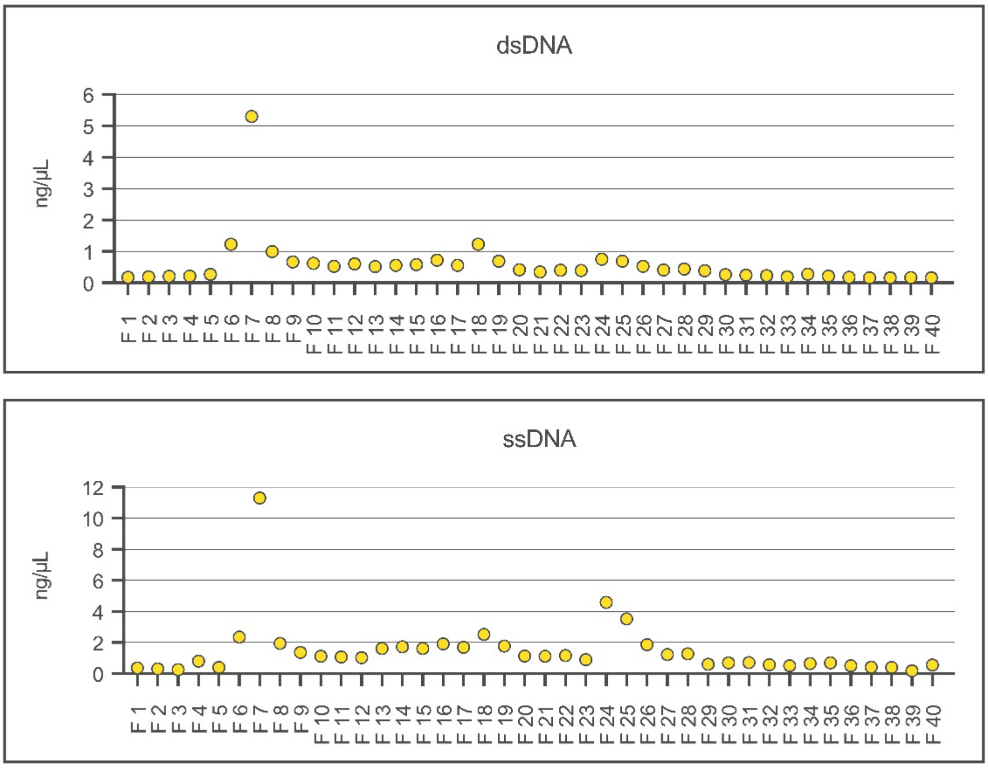

Supplementary Figure 1: Quantification of double-stranded DNA (dsDNA) and single-stranded DNA (ssDNA) in fractions (F) 1-40. The content of dsDNA and ssDNA in each fraction was quantified by dye-assisted fluorimetry with a Quantus^{TM} (Promega.com). Concentrations of dsDNA (upper panel) and ssDNA (lower panel) are given in ng/µL.

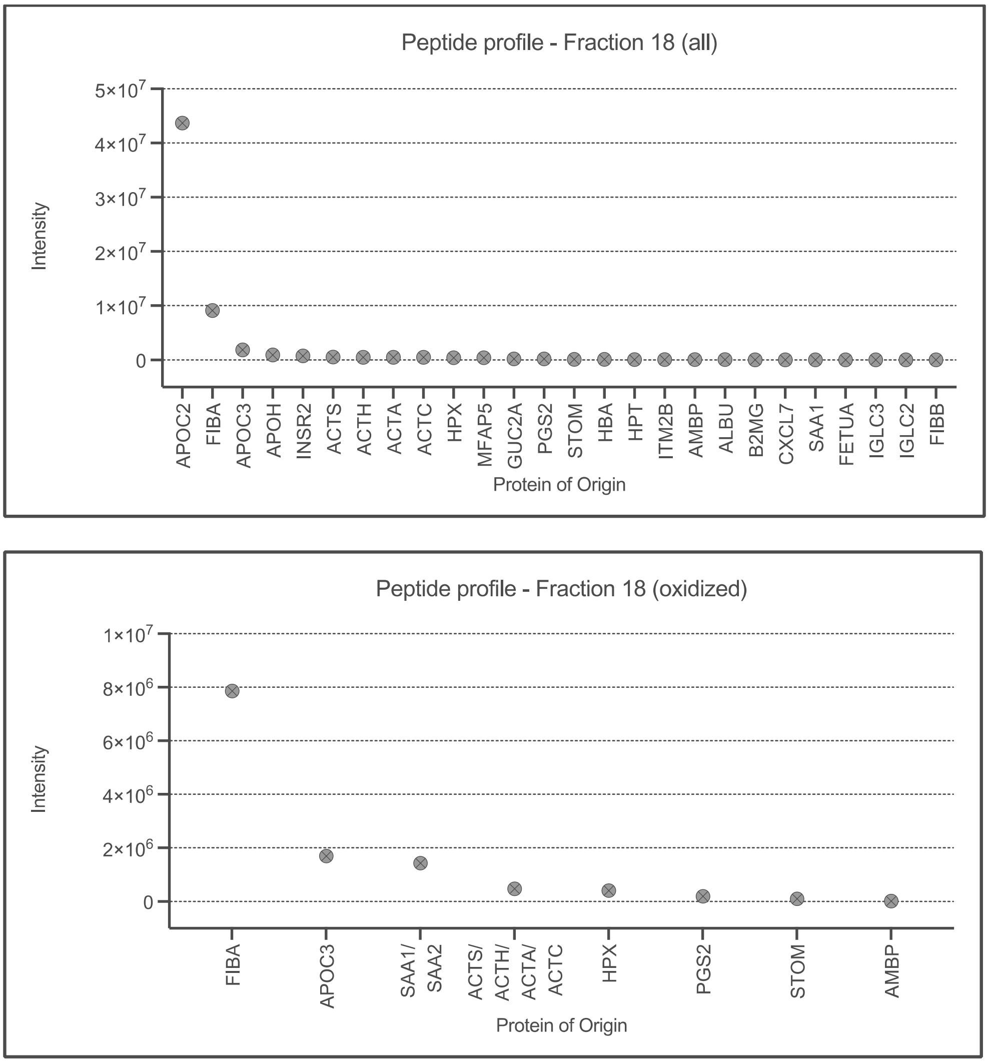

Supplementary Figure 2: Peptide profile of fraction 18 examined via mass spectrometry. Twenty-six peptides were found (post-translationally modified and non-modified) and are displayed in descending order of amount (peptide ranks). Quantity is registered in the intensity of absorbance on the chromatogram. Additionally found oxidized forms of the containing peptides are displayed in the diagram below. APOC2: Apolipoprotein C-II; FIBA: Fibrinogen Alpha Chain; APOC3: Apolipoprotein C-III; APOH: Beta-2-Glycoprotein 1; INSR2: Insulin Isoform 2; ACTS: Actin, Alpha Skeletal Muscle; ACTH: Actin, Gamma-Enteric Smooth Muscle; ACTA: Actin, Aortic Smooth Muscle; ACTC: Actin, Alpha Cardiac Muscle; HPX: Hemopexin; MFAP5: Microfibrillar-Associated Protein 5; GUC2A: Guanylin; PGS2: Decorin; STOM: Erythrocyte Band 7 Integral Membrane Protein; HBA: Hemoglobin Subunit Alpha; HPT: Haptoglobin; ITM2B: Integral Membrane Protein 2B; AMBP: Alpha-1 Microglycoprotein; ALBU: Serum Albumin; B2MG: Beta-2-Microglobulin; CXCL7: Platelet Basic Protein; SAA1/2: Serum Amyloid A-1/2 Protein; FETUA: Fetuin A, Alpha-2-HS-Glycoprotein; IGLC2/IGLC3: Immunoglobulin Lambda Constant 2/3; FIBB: Fibrinogen Beta Chain.

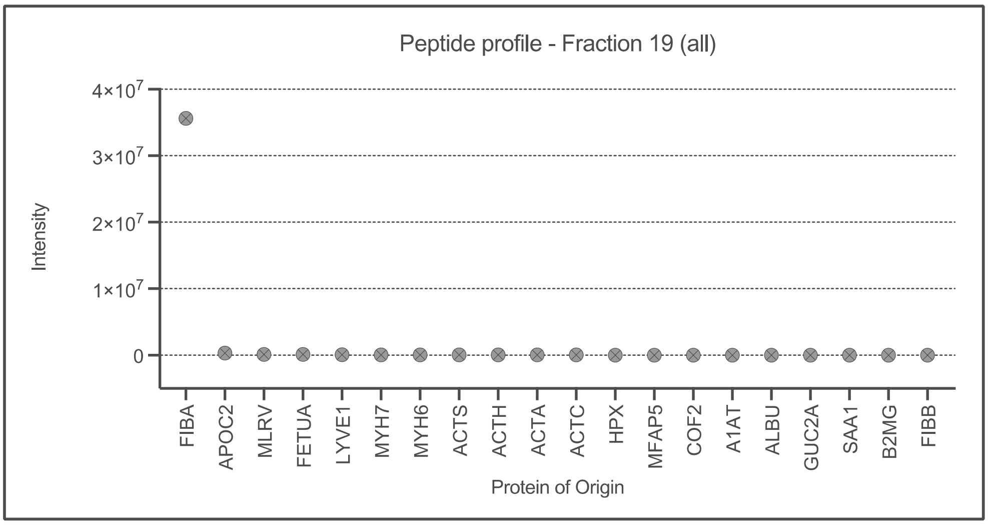

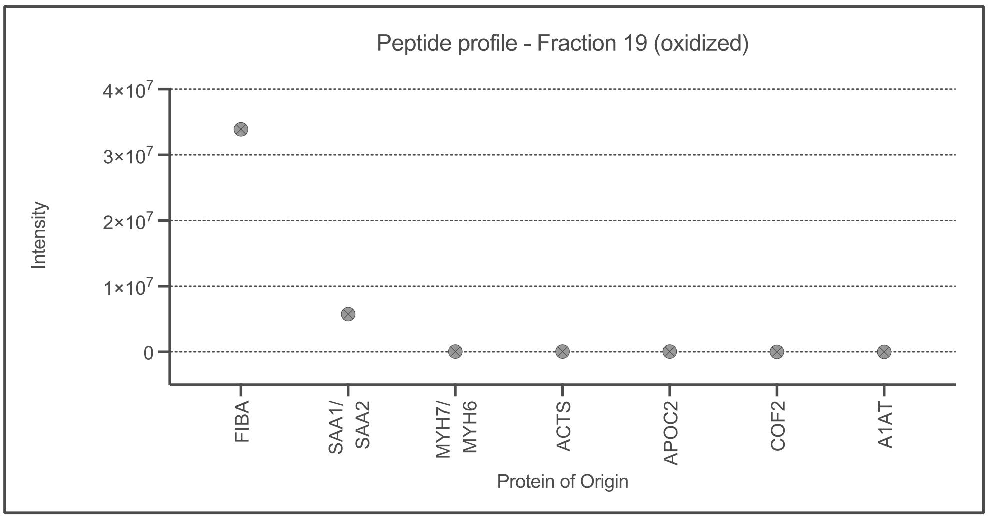

Supplementary Figure 3: Peptide profile of fraction 19 examined via mass spectrometry. Twenty peptides were found (post-translationally modified and non-modified) and are displayed in descending order of amount (peptide ranks). Quantity is registered in the intensity of absorbance on the chromatogram. Additionally found oxidized forms of the containing peptides are displayed in the diagram below. FIBA: Fibrinogen Alpha Chain; APOC2: Apolipoprotein C-II; MLRV: Myosin Regulatory Light Chain 2; FETUA: Fetuin A, Alpha-2-HS-Glycoprotein; LYVE1: Lymphatic Vessel Endothelial Hyaluronic Acid Receptor 1; MYH6/7: Myosin-6/7; ACTS: Actin, Alpha Skeletal Muscle; ACTH: Actin, Gamma- Enteric Smooth Muscle; ACTA: Actin, Aortic Smooth Muscle; ACTC: Actin, Alpha Cardiac Muscle; HPX: Hemopexin; MFAP5: Microfibrillar-Associated Protein 5; COF2: Cofilin-2; A1AT: Alpha-1- Antitrypsin; ALBU: Serum Albumin; GUC2A: Guanylin; SAA1/2: Serum Amyloid A-1/2 Protein; B2MG: Beta-2-Microglobulin; FIBB: Fibrinogen Beta Chain.

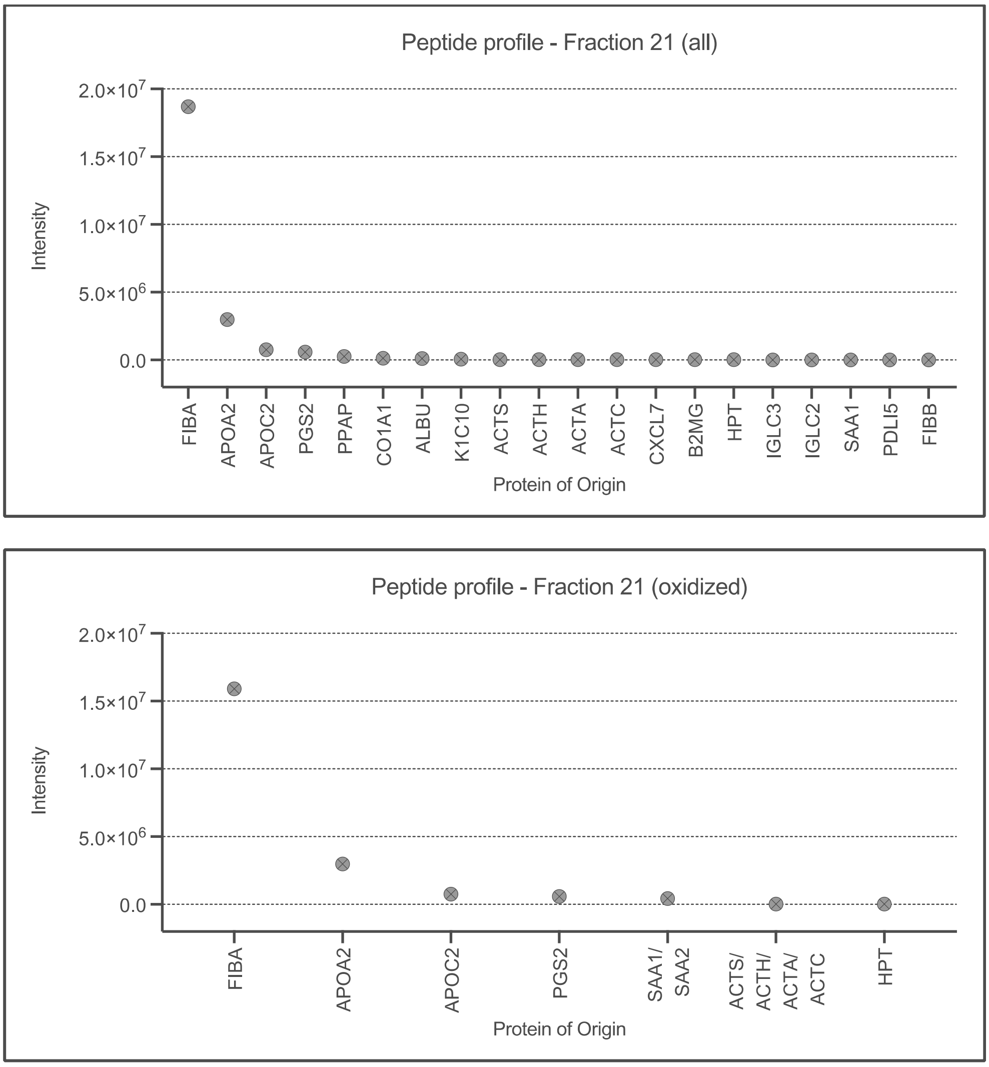

Supplementary Figure 4: Peptide profile of fraction 21 examined via mass spectrometry. Twenty peptides were found (post-translationally modified and non-modified) and are displayed in descending order of amount (peptide ranks). Quantity is registered in the intensity of absorbance on the chromatogram. Additionally found oxidized forms of the containing peptides are displayed in *the* diagram below. FIBA: Fibrinogen Alpha Chain; APOA2: Apolipoprotein A-II; APOC2: Apolipoprotein C-II; PGS2: Decorin; PPAP: Prostatic Acid Phosphatase; CO1A1: Collagen Alpha-1(I) Chain; ALBU: Serum Albumin; K1C10: Keratin, Type I Cytoskeletal 10; ACTS: Actin, Alpha Skeletal Muscle; ACTH: Actin, Gamma-Enteric Smooth Muscle; ACTA: Actin, Aortic Smooth Muscle; ACTC: Actin, Alpha Cardiac Muscle; CXCL7: Platelet Basic Protein; B2MG: Beta-2-Microglobulin; HPT: Haptoglobin; IGLC2/IGLC3: Immunoglobulin Lambda Constant 2/3; SAA1/2: Serum Amyloid A-1/2 Protein; PDLI5: PDZ And LIM Domain Protein 5; FIBB: Fibrinogen Beta Chain.

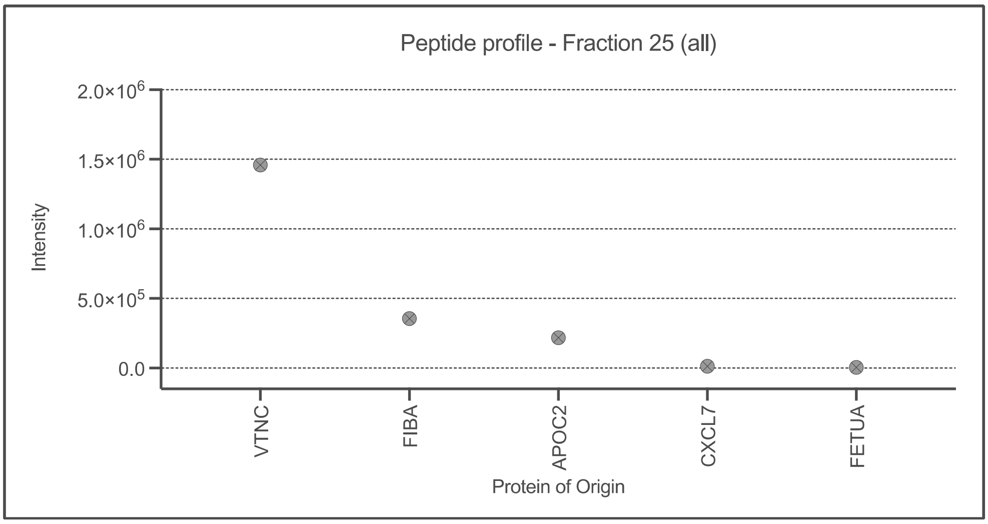

Supplementary Figure 5: Peptide profile of fraction 25 examined via mass spectrometry. Five peptides were found (post-translationally modified and non-modified) and are displayed in descending order of amount (peptide ranks). Quantity is registered in the intensity of absorbance on the chromatogram. Additionally found oxidized forms of the containing peptides are displayed in *the* diagram below. VTNC: Vitronectin; FIBA: Fibrinogen Alpha Chain; APOC2: Apolipoprotein C-II; CXCL7: Platelet Basic Protein; FETUA: Fetuin A, Alpha-2-HS-Glycoprotein.

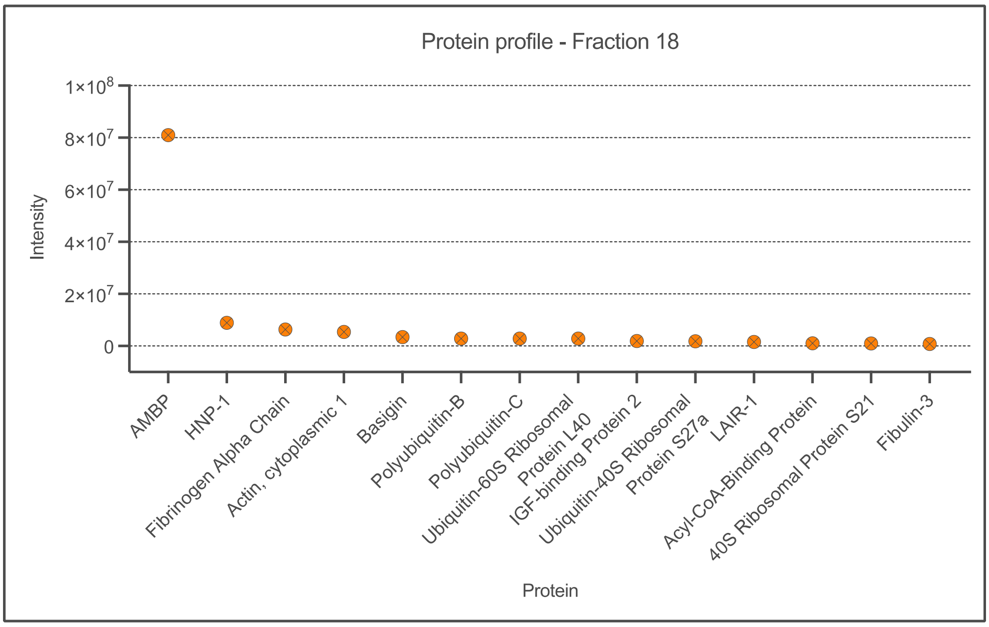

Supplementary Figure 6: Protein profile of fraction 18 determined by mass spectrometry. Due to the large number of proteins found in each fraction, only those proteins exceeding 1% of the maximum intensity are shown. The respective intensity in the chromatogram is shown in descending order. On the x-axis, the found proteins are presented in the form of the gene coding for them as well as their involved pathway according to the Pathway Commons Database. P02760|AMBP: AMBP (Alpha-1-Microglobulin/Bikunin Precursor); P59665|DEF1: Neutrophil Defensin 1, HNP-1; P02671|FIBA: Fibrinogen Alpha Chain; P60709|ACTB: Actin, Cytoplasmic 1; P35613|BASI: Basigin; P0CG47|UBB: Polyubiquitin-B; P0CG48|UBC: Polyubiquitin-C; P62987|RL40: Ubiquitin-60S Ribosomal Protein L40; P18065|IBP2: Insulin-Like Growth Factor-Binding Protein 2; P62979|RS27A: Ubiquitin-40S Ribosomal Protein S27a; Q6GTX8|LAIR1: Leukocyte-Associated Immunoglobulin-Like Receptor 1; P07108|ACBP: Acyl-CoA-Binding Protein; P63220|RS21: 40S Ribosomal Protein S21; Q12805|FBLN3: EGF- Containing Fibulin-Like Extracellular Matrix Protein 1.

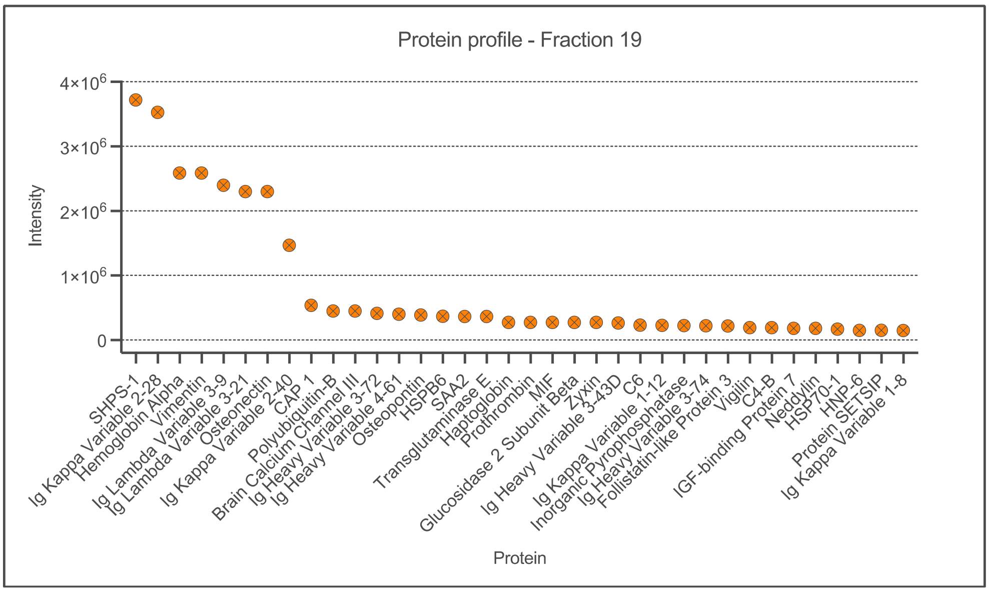

Supplementary Figure 7: Protein profile of fraction 19 determined by mass spectrometry. Due to the large number of proteins found in each fraction, only those proteins exceeding 4% of the maximum intensity are shown. The respective intensity in the chromatogram is shown in descending order. On the x-axis, the found proteins are presented in the form of the gene coding for them as well as their involved pathway according to the Pathway Commons Database. P78324|SHPS1: Tyrosine-Protein Phosphatase Non-Receptor Type Substrate 1; A0A075B6P5|KV228: Immunoglobulin Kappa Variable 2-28; P69905|HBA: Hemoglobin Subunit Alpha; P08670|VIME: Vimentin; A0A075B6K5|LV39: Immunoglobulin Lambda Variable 3-9; P80748|LV321: Immunoglobulin Lambda Variable 3-21; P09486|SPRC: SPARC; A0A087WW87|KV240: Immunoglobulin Kappa Variable 2-40; Q01518|CAP1: Adenylyl Cyclase-Associated Protein 1; P0CG47|UBB: Polyubiquitin-B; Q00975|CAC1B: Voltage-Dependent N-Type Calcium Channel Subunit Alpha-1B; A0A0B4J1Y9|HV372: Immunoglobulin Heavy Variable 3-72; A0A0C4DH41|HV461: Immunoglobulin Heavy Variable 4-61; P10451|OSTP: Osteopontin; O14558|HSPB6: Heat Shock Protein Beta-6; P0DJI9|SAA2: Serum Amyloid A-2 Protein; Q08188|TGM3: Protein-Glutamine Gamma-Glutamyltransferase E; P00738|HPT: Haptoglobin; P00734|THRB: Prothrombin; P14174|MIF: Macrophage Migration Inhibitory Factor; P14314|GLU2B: Glucosidase 2 Subunit Beta; Q15942|ZYX: Zyxin; P0DP04|HV43D: Immunoglobulin Heavy Variable 3-43D; P13671|CO6: Complement Component C6; A0A0C4DH73|KV112: Immunoglobulin Kappa Variable 1-12; Q15181|IPYR: Inorganic Pyrophosphatase; A0A0B4J1X5|HV374: Immunoglobulin Heavy Variable 3-74; O95633|FSTL3: Follistatin-Related Protein 3; Q00341|VIGLN: Vigilin; P0C0L5|CO4B: Complement C4-B; Q16270|IBP7: Insulin-Like Growth Factor-Binding Protein 7; Q15843|NEDD8: NEDD8; P0DMV8|HS71A: Heat Shock 70 kDa Protein 1A; Q01524|DEF6: Defensin-6; P0DME0|SETLP: Protein SETSIP; A0A0C4DH67|KV108: Immunoglobulin Kappa Variable 1-8.

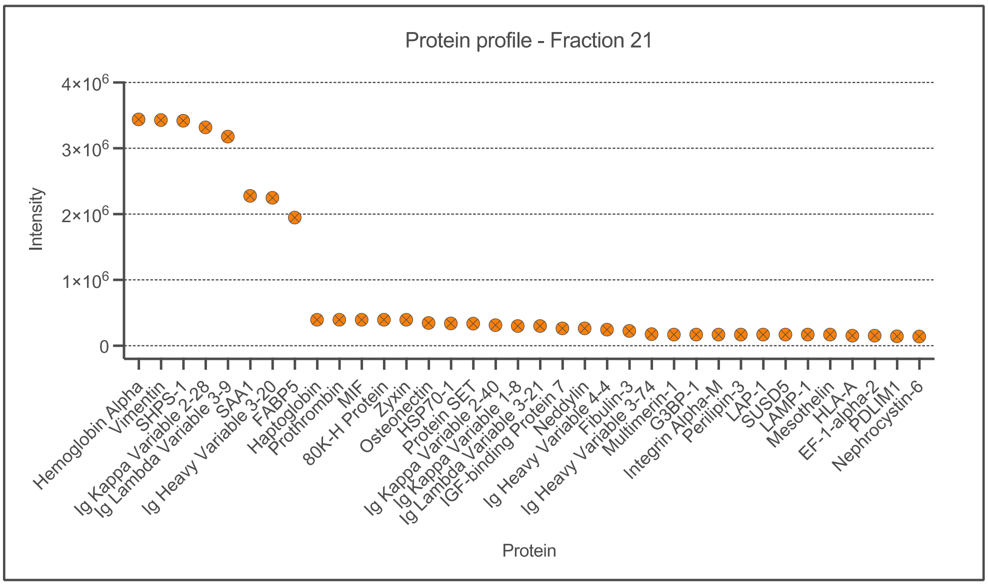

Supplementary Figure 8: Protein profile of fraction 21 determined by mass spectrometry. Due to the large number of proteins found in each fraction, only those proteins exceeding 4% of the maximum intensity are shown. The respective intensity in the chromatogram is shown in descending order. On the x-axis, the found proteins are presented in the form of the gene coding for them as well as their involved pathway according to the Pathway Commons Database. P69905|HBA: Hemoglobin Subunit Alpha; P08670|VIME: Vimentin; P78324|SHPS1: Tyrosine- Protein Phosphatase Non-Receptor Type Substrate 1; A0A075B6P5|KV228: Immunoglobulin Kappa Variable 2-28; A0A075B6K5|LV39: Immunoglobulin Lambda Variable 3-9; P0DJI8|SAA1: Serum Amyloid A-1 Protein; A0A0C4DH32|HV320: Immunoglobulin Heavy Variable 3-20; Q01469|FABP5: Fatty Acid-Binding Protein 5; P00738|HPT: Haptoglobin; P00734|THRB: Prothrombin; P14174|MIF: Macrophage Migration Inhibitory Factor; P14314|GLU2B: Glucosidase 2 Subunit Beta; Q15942|ZYX: Zyxin; P09486|SPRC: SPARC; P0DMV8|HS71A: Heat Shock 70 kDa Protein 1A; Q01105|SET: Protein SET; A0A087WW87|KV240: Immunoglobulin Kappa Variable 2-40; A0A0C4DH67|KV108: Immunoglobulin Kappa Variable 1-8; P80748|LV321: Immunoglobulin Lambda Variable 3-21; Q16270|IBP7: Insulin-Like Growth Factor-Binding Protein 7; Q15843|NEDD8: Neddylin; A0A075B6R2|HV404: Immunoglobulin Heavy Variable 4-4; Q12805|FBLN3: EGF-Containing Fibulin-Like Extracellular Matrix Protein 1; A0A0B4J1X5|HV374: Immunoglobulin Heavy Variable 3-74; Q13201|MMRN1: Multimerin-1; Q13283|G3BP1: Ras GTPase-Activating Protein-Binding Protein 1; P11215|ITAM: Integrin Alpha- M; O60664|PLIN3: Perilipin-3; P11142|HSP7C: Heat Shock Cognate 71 kDa Protein; O60279|SUSD5: Sushi Domain-Containing Protein 5; P11279|LAMP1: Lysosome-Associated Membrane Glycoprotein 1; Q13421|MSLN: Mesothelin; P10316|1A69: HLA Class I Histocompatibility Antigen, A Alpha Chain; Q05639|EF1A2: Elongation Factor 1-Alpha 2; O00151|PDLI1: PDZ And LIM Domain Protein 1; O15078|CE290: Centrosomal Protein Of 290 kDa, Nephrocystin-6.

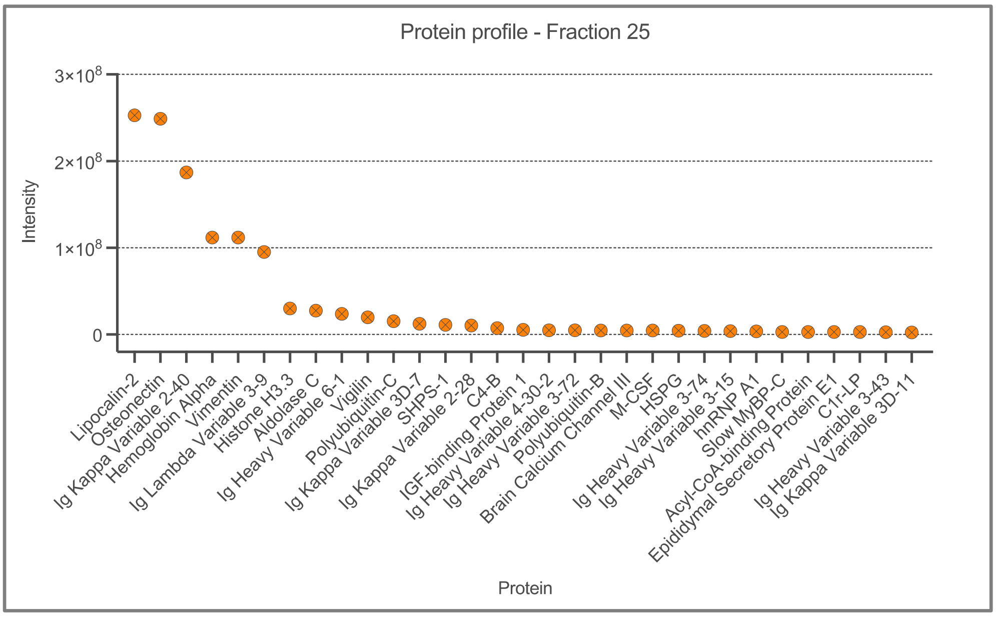

Supplementary Figure 9: Protein profile of fraction 25 determined by mass spectrometry. Due to the large number of proteins found in each fraction, only those proteins exceeding 1% of the maximum intensity are shown. The respective intensity in the chromatogram is shown in descending order. On the x-axis, the found proteins are presented in the form of the gene coding for them as well as their involved pathway according to the Pathway Commons Database. P80188|NGAL: Neutrophil Gelatinase-Associated Lipocalin; P09486|SPRC: SPARC; A0A087WW87|KV240: Immunoglobulin Kappa Variable 2-40; P69905|HBA: Hemoglobin Subunit Alpha; P08670|VIME: Vimentin; A0A075B6K5|LV39: Immunoglobulin Lambda Variable 3-9; P84243|H33: Histone H3.3; P09972|ALDOC: Fructose-Bisphosphate Aldolase C; A0A0B4J1U7|HV601: Immunoglobulin Heavy Variable 6-1; Q00341|VIGLN: Vigilin; P0CG48|UBC: Polyubiquitin-C; A0A0C4DH55|KVD07: Immunoglobulin Kappa Variable 3D-7; P78324|SHPS1: Tyrosine-Protein Phosphatase Non-Receptor Type Substrate 1; A0A075B6P5|KV228: Immunoglobulin Kappa Variable 2-28; P0C0L5|CO4B: Complement C4-B; P08833|IBP1: Insulin-Like Growth Factor-Binding Protein 1; A0A087WSY4|HV432: Immunoglobulin Heavy Variable 4-30-2; A0A0B4J1Y9|HV372: Immunoglobulin Heavy Variable 3-72; P0CG47|UBB: Polyubiquitin-B; Q00975|CAC1B: Voltage-Dependent N-Type Calcium Channel Subunit Alpha-1B; P09603|CSF1: Macrophage Colony- Stimulating Factor 1; P98160|PGBM: Basement Membrane-Specific Heparan Sulfate Proteoglycan Core Protein; A0A0B4J1X5|HV374: Immunoglobulin Heavy Variable 3-74; A0A0B4J1V0|HV315: Immunoglobulin Heavy Variable 3-15; P09651|ROA1: Heterogeneous Nuclear Ribonucleoprotein A1; Q00872|MYPC1: Myosin- Binding Protein C, Slow-Type; P07108|ACBP: Acyl-CoA-Binding Protein; P61916|NPC2: NPC Intracellular Cholesterol Transporter 2; Q9NZP8|C1RL: Complement C1r Subcomponent-Like Protein; A0A0B4J1X8|HV343: Immunoglobulin Heavy Variable 3-43; A0A0A0MRZ8|KVD11: Immunoglobulin Kappa Variable 3D-11.

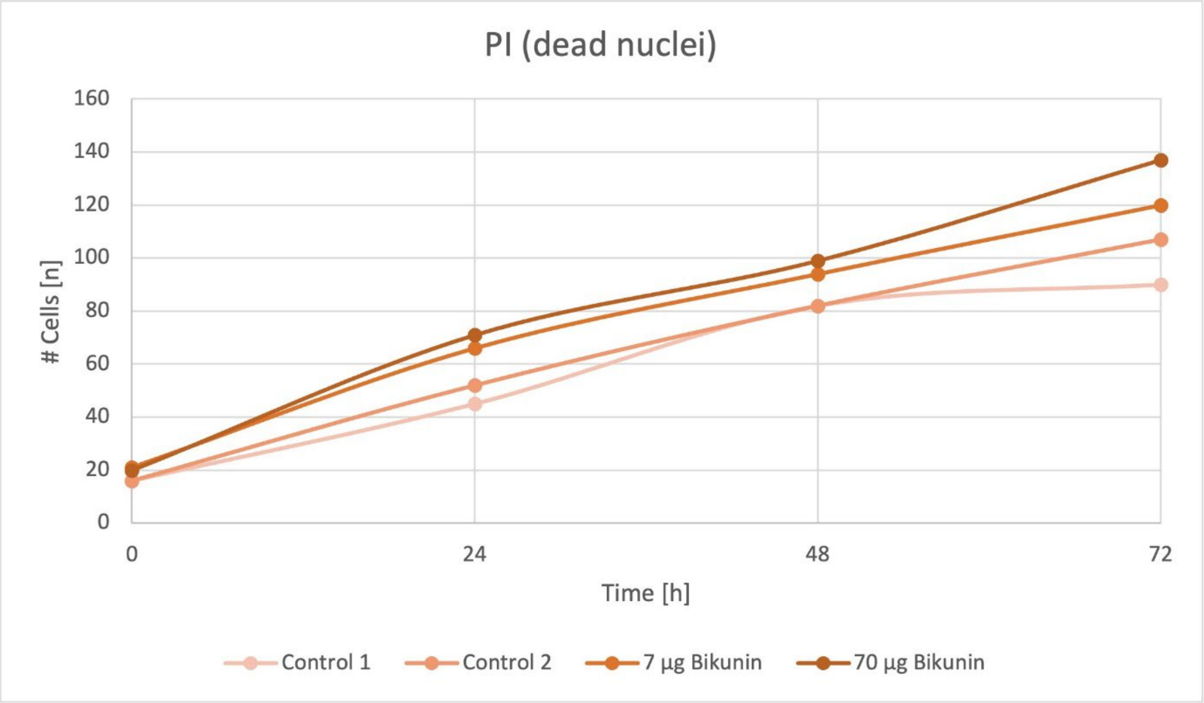

Supplementary Figure 10: Concentration dependent cell death occured in Ea.hy926 cells when cultured for 72 hours (h) in the presence of bikunin 7 µg/mL and 70 µg/mL isolated from human urine (left). Dead cells (n) were stained with propidium iodide (PI). The bikunin preparation used in these experiments was a kind gift from Dr. Jan Lawrenz (Molecular Virology, Ulm University).

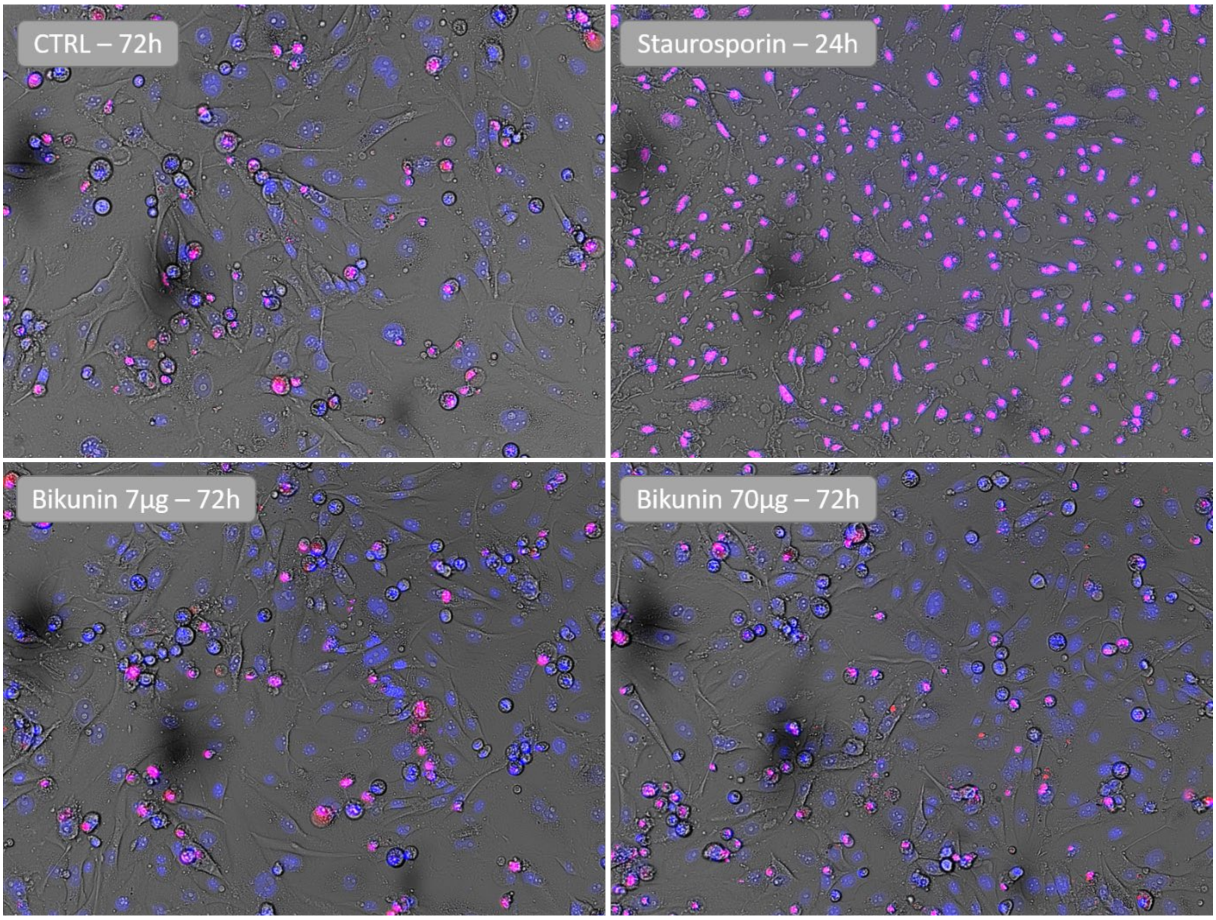

Supplementary Figure 11: Data analysis of dead Ea.hy926 cells during 72 h of observation in the absence or presence of bikunin 7 µg/mL and 70 µg/ml. For the positive control staurosporine 100 nM was used. Nuclei were stained with Hoechst (blue), dead cells were stained with propidium iodide (pink).

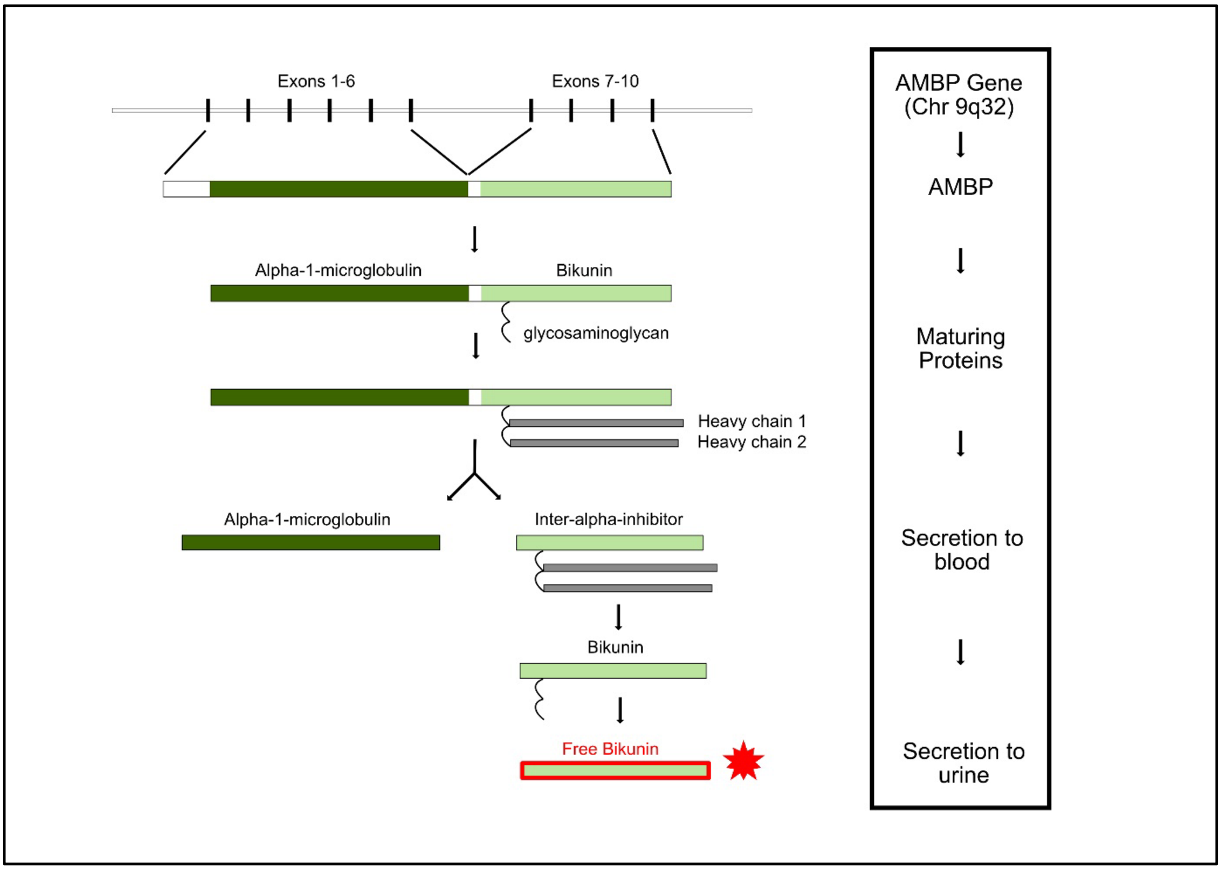

Supplementary Figure 12: Schematic of bikunin synthesis. Bikunin is mostly synthesized in the liver from the AMBP protein (352 amino acids), which also bears alpha-1-microglobulin. Maturation is associated with proteolytic cleavage of alpha-1-microglobulin and bikunin. The latter is then modified by glycosylation in the Golgi apparatus and further linked to heavy chains through an ester bond with a non-sulfated N-acetylgalactosamine (GalNAc) residue of the chodroitin sulfate chain. This molecule is released to the plasma and further excreted in the urine after loosing the heavvy chains and the glycosaminoglycan chain respectively. According to our hypothesis free bikunin may play a vital role in endothelial damage.

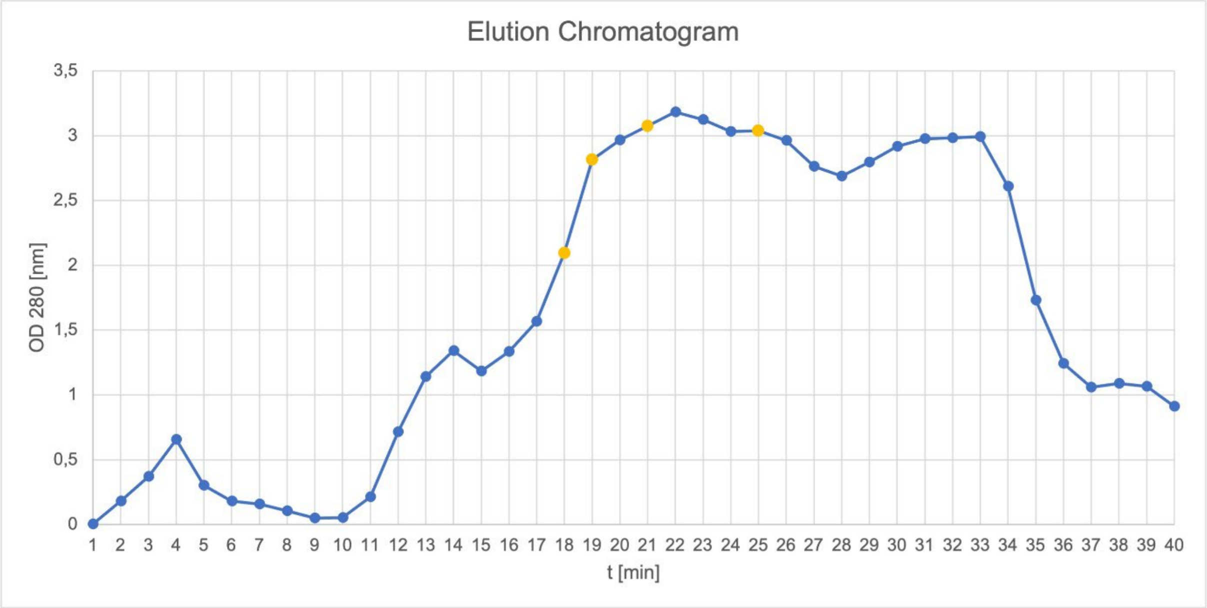

Supplementary Figure 13: Chormatographic profile of the 40 obtained protein fractions from Cytosorb hemadsorption filters (For detailed methods we refer to the materials and methods section in the manuscript. Shown is the elution chromatogram of the pooled Cytosorb filters. On the y-axis the absorbance (OD, optical density) at 280 nm is shown. The x-axis shows the distribution of the 40 fractions over time (t) in minutes (min). Fractions of interest are marked in yellow.

Supplementary Table 1: Overall protein concentrations in mg/mL in Cytosorb fractions exhibiting endothelial damage (#17, #18, 21), or not (#19, #25).

**Cystosorb fraction**	**Concentration [mg/mL]**
17	2,33674
18	3,12806
19	4,20254
21	4,58593
25	4,53204

Supplementary Tables 2-6: Protein concentrations in mg/mL in Cytosorb fractions exhibiting endothelial damage (#17, #18, 21), or not (#19, #25). AMBP: Alpha-1-Microglobulin/Bikunin Precursor; HNP-1/3: Neutrophil Defensin 1/3; SHPS1: Tyrosine-Protein Phosphatase; CAP1: Adenylyl Cyclase-Associated Protein 1; SAA1: Serum Amyloid A-1 Protein; FABP5: Fatty Acid-Binding Protein 5; MIF: Macrophage Migration Inhibitory Factor; 80K-H Protein: Glucosidase 2 Subunit Beta.

Supplementary Table 2: Fraction #17 (causing cell death)

**Protein**	**Concentration [mg/mL]**
AMBP	0.85174
Fibrinogen Alpha Chain	0.15901
Albumin	0.14905
Leukocyte-associated immunoglobulin-like receptor 1	0.10177
Proline-rich acidic protein 1	0.09983
Dermcidin	0.07439
EGF-containing fibulin-like extracellular matrix protein 1	0.05918
Immunoglobulin heavy constant alpha 1	0.03153
Complement factor B	0.02959
HNP-3	0.02904
HNP-1	0.02904
Arginase-1	0.02876
Hornerin	0.02702
Caspase-14	0.02528
Multimerin-1	0.02450
Histone H2B type 1-B	0.02370
Others	

Supplementary Table 3: Fraction #18 (causing cell death)

**Protein**	**Concentration [mg/mL]**
AMBP	1.758931
HNP-1	0.193700
Fibrinogen Alpha Chain	0.137457
Actin, cytoplasmic 1	0.118131
Basigin	0.075786
Polyubiquitin-B	0.061454
Polyubiquitin-C	0.061454
Ubiquitin-60S Ribosomal Protein L40	0.061454
IGF-binding Protein 2	0.041693
Ubiquitin-40S Ribosomal Protein S27a	0.039305
Leukocyte-associated immunoglobulin-like receptor 1	0.034310
Others	

Supplementary Table 4: Fraction #19 (causing cell death)

Protein	Concentration [mg/mL]
SHPS-1	0.46487
Ig Kappa Variable 2-28	0.44113
Hemoglobin Alpha	0.32366
Vimentin	0.32366
Ig Lambda Variable 3-9	0.29992
Ig Lambda Variable 3-21	0.28742
Osteonectin	0.28742
Ig Kappa Variable 2-40	0.18370
CAP 1	0.06723
Polyubiquitin-B	0.05648
Brain Calcium Channel III	0.05623
Ig Heavy Variable 3-72	0.05186
Others	

Supplementary Table 5: Faction #21 (causing minor cell death)

Protein	Concentration [mg/mL]
Hemoglobin Alpha	0.43805
Vimentin	0.43677
SHPS-1	0.43550
Ig Kappa Variable 2-28	0.42277
Ig Lambda Variable 3-9	0.40494
SAA1	0.29033
Ig Heavy Variable 3-20	0.28651
FABP5	0.24831
Haptoglobin	0.05043
Prothrombin	0.05043
MIF	0.05043
80K-H Protein	0.05043
Zyxin	0.05043
Others	

Supplementary Table 6: Fraction #25 (inactive)

Protein	Concentration [mg/mL]
Lipocalin-2	0.90704
Osteonectin	0.89269
Ig Kappa Variable 2-40	0.67042
Hemoglobin Alpha	0.40153
Vimentin	0.40153
Ig Lambda Variable 3-9	0.34166
Histone H3.3	0.10755
Aldolase C	0.09895

Ig Heavy Variable 6-1	0.08568
Vigilin	0.07099
Polyubiquitin-C	0.05521
Others	
